# A Multicomponent Strategy to Increase Human Papillomavirus Vaccination Rates in Primary Care: A Cluster Randomized Clinical Trial

**DOI:** 10.1001/jamanetworkopen.2026.0049

**Published:** 2026-02-26

**Authors:** Ruoyun Wang, Lei Liu, Sherry Dodd, Sharon Graham, Shannon Rook, Lauren Ericson, Katie Plax, Abigail Barker, Virginia McKay, Michelle I. Silver, Jason G. Newland, Allison A. King

**Affiliations:** 1 Department of Pediatrics, Washington University School of Medicine, St Louis, Missouri; 2 Division of Biostatistics, Washington University School of Medicine, St Louis, Missouri; 3 Office of Medical Student Education, Washington University, St Louis, Missouri; 4 School of Public Health, Washington University, St Louis, Missouri; 5 Division of Public Health Sciences, Department of Surgery, Washington University School of Medicine, St Louis, Missouri; 6 Department of Pediatrics, Nationwide Children’s Hospital and The Ohio State University College of Medicine, Columbus

## Abstract

**Importance:**

Human papillomavirus (HPV) vaccination prevents multiple cancers, yet vaccination rates by the recommended age remain well below national goals.

**Objective:**

To evaluate the effectiveness of a multicomponent strategy in increasing HPV vaccination rates in primary care settings by 13 years of age.

**Design, Setting, and Participants:**

This cluster randomized clinical trial with a parallel-group design and 1:1 allocation was conducted at 20 pediatrics primary care practices from a practice-based research network in the St Louis, Missouri, metropolitan area by clinicians delivering routine well-child visits within participating practices. The intervention was implemented over 24 months (July 1, 2020, to September 30, 2022) with follow-up at 36 months (July 1, 2022, to September 30, 2023; 12 months after the intervention). Baseline characteristics were assessed using 2019 data.

**Interventions:**

(1) Practice facilitation by a trained external facilitator, (2) audit and feedback of HPV vaccination rates, (3) HPV vaccination education, and (4) announcement-style communication strategy.

**Main Outcomes and Measures:**

The primary outcomes were the proportion of patients who received the first HPV vaccine (initiation) and completed the series (completion) by 13 years of age, assessed at 24 months. Enduring effect was assessed at 36 months. Data were analyzed based on the intention-to-treat principle.

**Results:**

A total of 86 clinicians participated (40 control and 46 intervention; 65 women [75.6%]). Most were physicians (67 [77.9%]) and median time in practice was 18 years (IQR, 10-24 years). The intervention group had a higher percentage of privately insured patients than the control group (1562 of 1727 [90.4%] vs 717 of 1371 [52.3%]). At 24 months, HPV vaccination initiation rates were 80.1% (1723 of 2151) in the intervention group vs 75.5% (1219 of 1615) in the control group; completion rates were 52.8% (1135 of 2151) in the intervention group vs 50.1% (809 of 1615) in the control group. At 36 months, initiation rates were 82.0% (1812 of 2211) in the intervention group vs 74.6% (1052 of 1410) in the control group; completion rates were 57.8% (1279 of 2211) in the intervention group vs 49.9% (703 of 1410) in the control group. Fully adjusted models showed no differences for vaccination initiation (24 months: odds ratio [OR], 1.15 [95% CI, 0.78-1.70]; 36 months: OR, 1.42 [95% CI, 0.96-2.11]) or completion (24 months: OR, 0.96 [95% CI, 0.72-1.29]; 36 months: OR, 1.24 [95% CI, 0.92-1.67]), although intervention effects for initiation increased significantly over time (*P* = .04).

**Conclusions and Relevance:**

In this cluster randomized clinical trial of a multicomponent intervention in 20 practices, HPV vaccination rates by 13 years of age were not significantly different between groups, although intervention effects increased significantly over time for initiation of vaccination. System-focused interventions may require extended evaluation periods to demonstrate measurable improvements.

**Trial Registration:**

ClinicalTrials.gov Identifier: NCT04180462

## Key Points

**Question:**

Does a multicomponent strategy combining practice facilitation with clinician behavior change interventions increase human papillomavirus (HPV) vaccination rates by 13 years of age in primary care settings?

**Findings:**

In this cluster randomized clinical trial of 20 practices with 86 clinicians, between-group differences in HPV vaccination rates were not significant at individual time points. However, a significant pattern of increasing intervention effects over time was observed for initiation.

**Meaning:**

These findings suggest that interventions designed to build sustainable practice systems may require extended evaluation periods to demonstrate their effect.

## Teaser

This cluster randomized clinical trial evaluates the effectiveness of a multicomponent strategy combining practice facilitation with clinician behavior change interventions in increasing human papillomavirus vaccination rates in primary care settings.

## Introduction

Human papillomavirus (HPV) causes multiple cancers in women and men,^1,2,3,4^ accounting for approximately 37 800 new cancer cases annually in the US.^5^ Given that HPV vaccines are safe and effective in preventing HPV-related cancers,^1,2,6,7^ the Centers for Disease Control and Prevention (CDC) Advisory Committee on Immunization Practices (ACIP) recommends HPV vaccination as early as 9 years of age, with completion by 13 years of age.^4^ However, the HPV vaccination rate remains below the Healthy People 2030 goal of 80% coverage.^8^ In 2024, only 63% of adolescents aged 13 to 17 years were up to date with the vaccination series.^9^

Achieving optimal HPV vaccination coverage requires addressing barriers across multiple levels of the health care system. Clinician barriers to increasing HPV vaccination rates include limited knowledge of HPV vaccination guidelines, low confidence in addressing vaccine hesitancy, and suboptimal communication strategies.^10,11,12,13^ Practice-level barriers include workflow inefficiencies, insufficient staffing and resources, and limited practice-level feedback mechanisms on vaccination performance.^14,15^ Tailored interventions provide comprehensive support that address these multilevel challenges to vaccination uptake.

Guided by the Consolidated Framework for Implementation Research^16^ and the Theoretical Domain Framework,^17^ we systematically identified barriers and facilitators to HPV vaccination in pediatric practices.^10,18^ External practice facilitators were the central mechanism for delivering tailored, ongoing support to practices to increase HPV vaccination rates.^19,20,21^ This approach, combining clinician education,^22,23,24,25^ audit and feedback,^25,26,27,28^ and communication training,^24,26,29,30,31^ addresses the interconnected challenges while building practice capacity for sustainable improvement. The study objective was to evaluate this multicomponent intervention in increasing HPV vaccination rates by 13 years of age.

## Methods

### Study Design and Setting

This cluster randomized clinical trial with a parallel-group design was conducted in the St Louis, Missouri, metropolitan area to evaluate the effectiveness of a theory-based, multicomponent intervention in primary care practices, aiming to increase HPV vaccination rates over 24 months (July 1, 2020, to September 30, 2022). An additional evaluation occurred at month 36 (July 1, 2022, to September 30, 2023) to assess the enduring effect of the intervention benefit. We tracked HPV vaccination rates at baseline (2019), intervention (12 and 24 months), and after intervention (36 months) from January 1, 2019, to September 30, 2023. The study was approved by the institutional review board of Washington University in St Louis. Written informed consent was obtained from participating practices and clinicians. The requirement for individual patient informed consent was waived by the institutional review board because the study involved no more than minimal risk and used retrospective medical record data. Study protocol is provided in Supplement 1. This study followed the Consolidated Standards of Reporting Trials (CONSORT) reporting guideline.

### Recruitment

**Figure 1.**
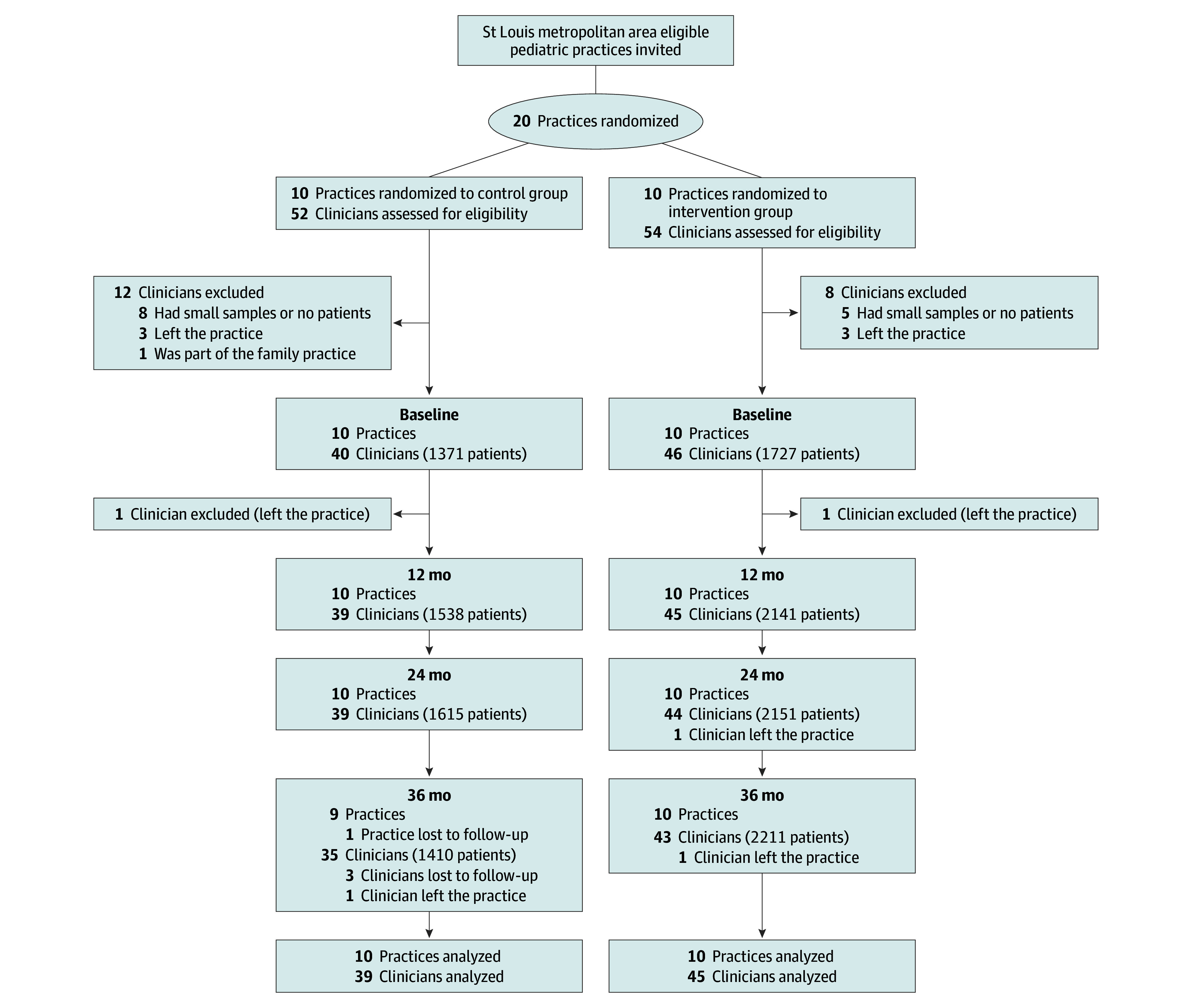
Flow Diagram of the Practice Clusters and Clinicians Baseline: 2019; intervention (12 months): July 2020 to June 2021 (group A) and October 2020 to September 2021 (group B); intervention (24 months): July 2021 to June 2022 (group A) and October 2021 to September 2022 (group B); postintervention (36 months): July 2022 to June 2023 (group A) and October 2022 to September 2023 (group B).

From November 1, 2019 to August 31, 2020, we contacted 49 pediatric practices associated with the Washington University Pediatric and Adolescent Ambulatory Research Consortium. The first 20 interested practices with available clinical staff were enrolled; solo-clinician practices were excluded. All clinicians delivering routine well-child visits were eligible for participation. We excluded clinicians with 10 or fewer patients at baseline in practices with more than 4 clinicians, as well as those who left the practice before the intervention commenced (Figure 1).

### Randomization

Practices were randomized 1:1 to control or intervention after baseline assessment, stratified by number of clinicians (≤4 or >4), using a block size of 2. A biostatistician (L.L.) blinded to practice identities conducted the randomization and remained masked throughout the subsequent data analysis. Clinicians and practice facilitators could not be blinded to assignment but were not given intervention details beforehand. Patients and families were unaware of their clinicians’ participation in training.

### Intervention

The intervention used clinician-focused behavioral strategies to support practice change.^18^ The components in this intervention included (1) practice facilitation by a trained external facilitator, (2) audit and feedback of vaccination rates, (3) HPV vaccination education, and (4) an announcement-style communication strategy. All components were integrated and delivered throughout the 24-month intervention.

Practice facilitation was the core component to assist practices and clinicians in implementing the change process, engaging staff, and addressing HPV vaccine barriers. Practice facilitators used quality improvement (QI) methods to support data-driven decision-making and develop practice-specific strategies. During this 24-month intervention, practice facilitators collaborated with the practice QI team (clinicians and clinical staff) to develop a sustainable HPV vaccine delivery system. Facilitators conducted semistructured interviews with QI teams, created workflows, and guided practices to develop at least 2 change ideas annually (eTable 1 in Supplement 2). Each practice chose the metrics to monitor the change and implemented a system for monitoring. Plan-Do-Study-Act^32^ cycles were used to discuss changes and address questions. Through this process, the QI team reviewed data at least every 6 months.

Audit and feedback reports were created from monthly data audits by practice facilitators to track change idea progress. Every 3 months, these reports, including practice-level and deidentified clinician-level data, were shared to encourage reflection and identify performance gaps.

HPV vaccination education included reviewing the ACIP guideline recommendations, explaining the age target rationale, and providing various educational resources such as American Cancer Society flyers,^33,34^ Washington University Pediatric and Adolescent Ambulatory Research Consortium brochures, a team-created educational video,^35^ and published peer-reviewed manuscripts. Posters and brochures were provided to practices for promoting HPV vaccination.

The announcement-style communication strategy^36^ was delivered by the facilitator to improve clinicians’ communication skills and self-efficacy in addressing parental hesitancy (eTable 2 in Supplement 2). Training included role playing and emphasized key elements of the communication strategy: making it routine, personal, and clear (eFigure 1 in Supplement 2).

**Figure 2.**
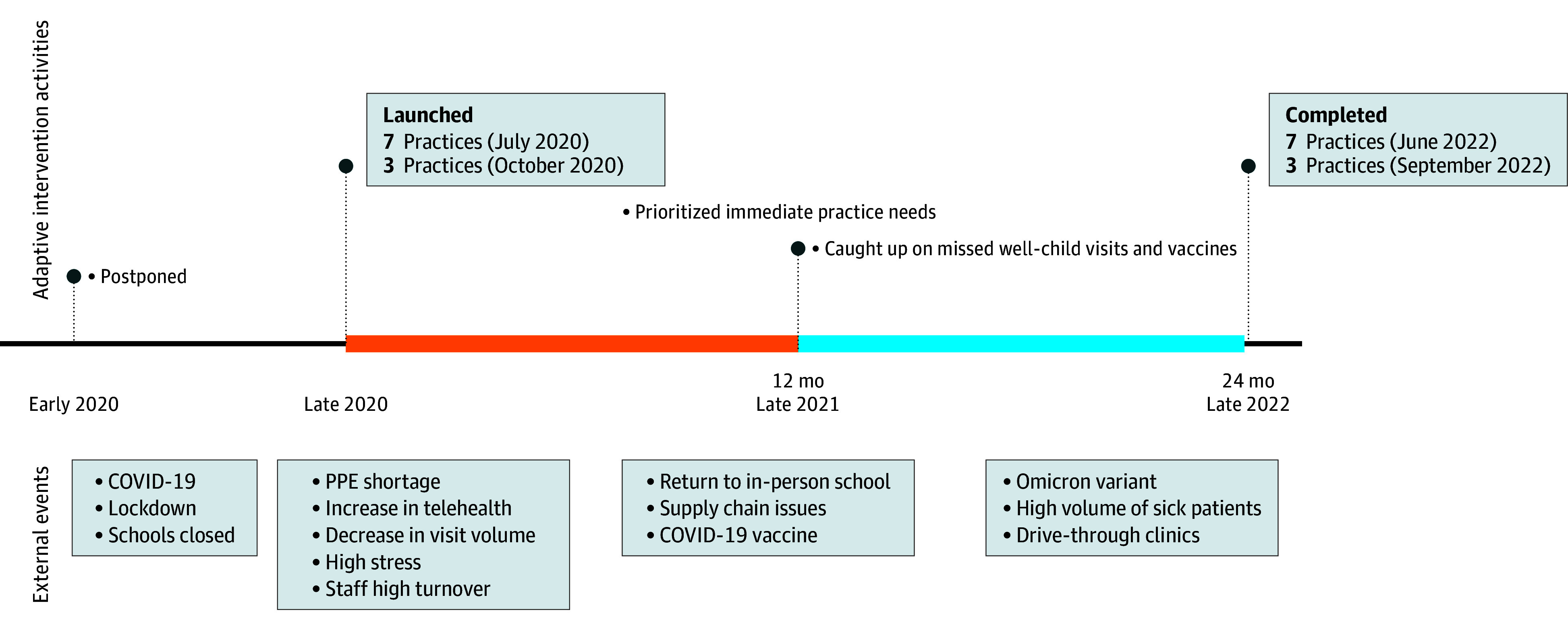
Timeline of Events During Intervention Period PPE indicates personal protective equipment.

The intervention start date was postponed due to the COVID-19 pandemic (Figure 2). Seven practices began in July 2020 (group A), and 3 practices began in October 2020 (group B). The intervention duration remained identical for all practices. Given COVID-19 safety protocols, facilitation shifted primarily from in person to virtual, with 80% of practices conducting more than 75% of sessions via Zoom. Practices received a median of 22 facilitation sessions (range, 17-27 [IQR, 20-25]), with each meeting lasting less than 1 hour. Practices received $500 per year to compensate for study measurements. Clinicians were offered American Board of Pediatrics Maintenance of Certification Part 4 credit and $50 per year for completing study surveys. Practices in the control group received publicly available information about HPV vaccines representing usual care, including current recommendations and CDC patient educational materials.

### Sample Size Determination

Sample size was determined using a simulation that was carried out in R, version 4.0.0 (R Project for Statistical Computing), accounting for a 3-level hierarchical structure (patients, clinicians, and practices). Using a contemporaneous Missouri HPV vaccination rate of 40%^37^ and assuming 4 clinicians per practice, with 30 patients each, power calculations indicated that 10 practices per group provided more than 80% power to detect a 13–percentage point difference (odds ratio [OR], 1.69) in vaccination rate at 2-sided α = .05. To ensure robust power accounting for potential practice size variation, we increased power to 60 patients per clinician.

### Measures

#### Primary Outcome: HPV Vaccination Rates

The primary outcomes were the proportion of patients who received the first dose of HPV vaccine (initiation) and those who completed the second dose (completion) by the patient’s 13th birthday. These measures were consistent with national guidelines and quality performance measures at the time of study design, including the HEDIS (Healthcare Effectiveness Data and Information Set) Immunizations for Adolescents metric.^38^ Vaccination and demographic information, including race and ethnicity (Asian, Black, Hispanic, White, or other [American Indian or Alaska Native, Native Hawaiian or Pacific Islander, and multiracial]), were extracted from the electronic health record or through onsite medical record review at baseline and at 12, 24, and 36 months. Race and ethnicity data for patients were collected to characterize the study population and practice settings.

Eligible patients included those who turned 13 years of age during each 12-month measurement period and had at least 1 medical encounter, ensuring an opportunity for vaccination. For each practice, data from sampled patients of all eligible clinicians were included in the analyses. Up to 60 patients per clinician were randomly sampled at each time point; if a clinician had fewer than 60 eligible patients, all were included. We compared the odds of HPV vaccination by 13 years of age between groups at each time point and over the study period.

#### Secondary Outcome: Clinician Knowledge and Practice

Clinicians were invited to complete a survey through Research Electronic Data Capture (REDCap)^39,40^ at baseline, 12 months, and 24 months to assess their HPV vaccination knowledge and practices; demographic information, including race and ethnicity (Asian, Black, Hispanic, White, or other [American Indian or Alaska Native, Native Hawaiian or Pacific Islander, and multiracial]), was collected at baseline. Race and ethnicity data for clinicians were collected to characterize the study population and practice settings. We assessed the frequency of use of the announcement-style communication (1 = never to 5 = always) and self-efficacy in addressing parental hesitancy (1 = strongly disagree to 7 = strongly agree). Both factors were highlighted in the multicomponent intervention strategy and were identified as affecting HPV vaccination in a previous study.^41^

### Statistical Analysis

Descriptive statistics on patient and clinician characteristics, survey questions, and unadjusted HPV vaccination rates were reported using the full sample. We imputed missing race values based on each practice’s racial distribution. Other covariates with missing values (2%) were removed from multivariable analyses.

We performed multilevel mixed-effects logistic regression analyses, modeling the status of HPV vaccination initiation (or completion) as a binary outcome (yes or no) for each patient. We first performed an unadjusted analysis including data from all time points and a group-by-time interaction term. Two sequential adjusted models used data from 12, 24, and 36 months; model 1 adjusted for clinician baseline HPV vaccination rate, and model 2 additionally adjusted for patient sex, race, insurance type, and clinician sex. Random effects accounted for clustering at the clinician and practice levels. Prespecified subgroup analyses examined patient sex and insurance type as moderators; race and clinician sex were exploratory.

For the clinician survey, we performed a linear mixed-effects model to evaluate the effect of the intervention on 2 key behavioral outcomes: (1) clinicians’ frequency of using an announcement-style communication and (2) self-efficacy of addressing parental hesitancy. To address potential nonresponse bias due to differential response rates, we compared clinician characteristics between survey responders and nonresponders within each group. Models were adjusted for baseline survey responses and clinician characteristics, with practice included as a random effect.

All tests were 2-sided with *P* < .05 considered statistically significant, and results were presented with 95% CIs or *P* values. Data were analyzed based on the intention-to-treat principle using SAS, version 9.4 (SAS Institute Inc).

## Results

All 20 practices participated throughout the 24-month intervention, and 1 control practice failed to provide data at 36 months (Figure 1). Detailed counts of clinicians and patients at each practice are provided in eTable 3 in Supplement 2. The control and intervention groups were comparable regarding metropolitan status, median number of clinicians, and baseline rates of HPV vaccination initiation and completion (Table 1).

**Table 1.** Baseline Characteristics of Practices, Clinicians, and Patients

Characteristic	No. (%)	
	Control	Intervention
Practices, No.	10	10
No. of clinicians per practice, median (IQR)	3 (2-5)	4 (4-5)
Location		
Metropolitan	10 (100.0)	9 (90.0)
Nonmetropolitan	0	1 (10.0)
HPV vaccination among patients by age 13 y, No./total No. (%)		
Initiation (1 dose)	946/1371 (69.0)	1234/1727 (71.5)
Completion (2 doses)	583/1371 (42.5)	750/1727 (43.4)
Clinicians, No.	40	46
Profession		
Physician	28 (70.0)	39 (84.8)
Nurse practitioner	11 (27.5)	7 (15.2)
Physician assistant	1 (2.5)	0
Sex		
Female	34 (85.0)	31 (67.4)
Male	6 (15.0)	15 (32.6)
Race		
Asian	4 (10.0)	2 (4.3)
Black	4 (10.0)	0
White	25 (62.5)	43 (93.5)
Other^a^	2 (5.0)	1 (2.2)
Missing	5 (12.5)	0
Ethnicity		
Hispanic or Latino	1 (2.5)	1 (2.2)
Non-Hispanic or non-Latino	31 (77.5)	41 (89.1)
Missing	8 (20.0)	4 (8.7)
Years in practice, median (IQR)	19 (10-24)	17 (10-23)
Announcement-style communication, mean (SD)^b^	3.28 (1.40)	3.34 (1.30)
Self-efficacy, mean (SD)^c^	5.38 (1.29)	5.50 (1.00)
Patients, No.	1371	1727
Sex		
Female	695 (50.7)	850 (49.2)
Male	676 (49.3)	877 (50.8)
Race		
Asian	14 (1.0)	42 (2.4)
Black	486 (35.5)	86 (5.0)
White	680 (49.6)	1172 (67.9)
Other^a^	45 (3.3)	39 (2.3)
Missing^d^	146 (10.7)	388 (22.5)
Ethnicity		
Hispanic or Latino	72 (5.3)	33 (1.9)
Non-Hispanic or non-Latino	1067 (77.8)	1288 (74.6)
Missing^d^	232 (16.9)	406 (23.5)
Insurance		
Private	717 (52.3)	1562 (90.4)
Medicaid	526 (38.4)	139 (8.1)
Self-pay	57 (4.2)	7 (0.4)
Missing^d^	71 (5.2)	19 (1.1)

Abbreviation: HPV, human papillomavirus.

^a^ Includes American Indian or Alaska Native, Native Hawaiian or Pacific Islander, and multiracial.

^b^ Announcement-style communication: how often did you use this approach when talking about HPV vaccination in the past 2 weeks? Response options: always (5), often (4), sometimes (3), rarely (2), and 1 (never).

^c^ Self-efficacy: delivering the vaccine according to guideline recommendations when parents are hesitant. Response options: strongly agree (7) to strongly disagree (1).

^d^ Missing information in health records due to practices not collecting the data or patients declining to disclose. Two intervention practices did not collect race and ethnicity across all study years. One control practice did not collect insurance at baseline.

Among the 86 clinicians (40 in the control group and 46 in the intervention groups; 65 women [75.6%] and 21 men [24.4%]; 6 Asian [7.0%], 4 Black [4.7%], 2 Hispanic or Latino [2.3%], 68 White [79.1%], 3 of other race or ethnicity [3.5%]), most were physicians (67 [77.9%]) (Table 1). Both groups included experienced clinicians who at baseline demonstrated comparable use of announcement styles and similar self-efficacy scores.

Baseline patient characteristics differed; the intervention group had a higher proportion of White patients, predominantly with private insurance, and a greater amount of missing race and ethnicity data compared with the control group (Table 1). These differences remained consistent throughout the study period.

### HPV Vaccination Rates by Study Period

**Figure 3.**
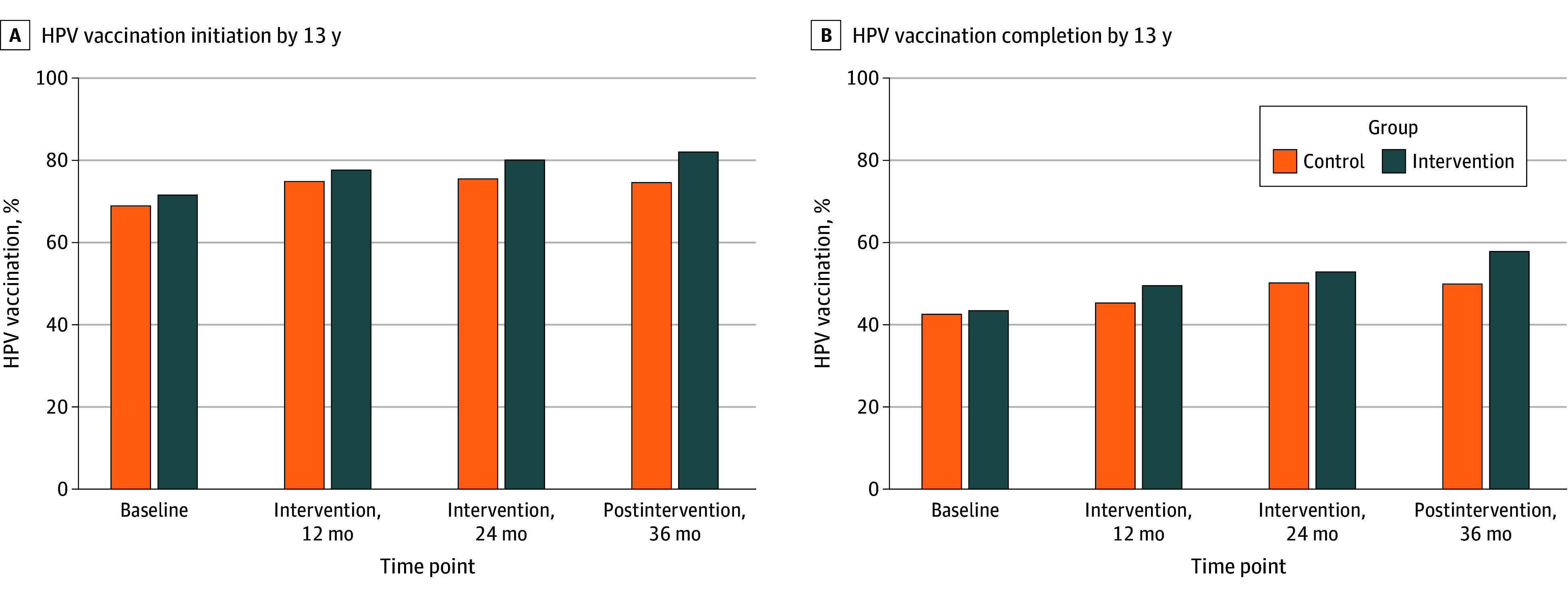
Unadjusted Human Papillomanvirus (HPV) Vaccination Initiation and Completion by Group A, HPV vaccination initiation by 13 years of age. B, HPV vaccination completion by 13 years of age. Data represent the unadjusted proportion of patients vaccinated at each time point. Time points represent months from intervention start. At 36 months, the control group included 9 practices (1 practice lost to follow-up). See Table 2 for adjusted analyses accounting for clustering and covariates.

The baseline vaccination initiation rate for both groups combined was 70.4% (2180 of 3098). HPV vaccination initiation by 13 years of age increased in both groups over 36 months (Figure 3). At 24 months, vaccination rates were 80.1% (1723 of 2151) in the intervention group vs 75.5% (1219 of 1615) in the control group. At 36 months, rates were 82.0% (1812 of 2211) in the intervention group vs 74.6% (1052 of 1410) in the control group. For HPV vaccination completion at 24 months, vaccination rates were 52.8% (1135 of 2151) in the intervention group vs 50.1% (809 of 1615) in the control group; at 36 months, completion rates were 57.8% (1279 of 2211) in the intervention group vs 49.9% (703 of 1410) in the control group.

### Intervention Effects on HPV Vaccination Rates

For HPV vaccination initiation, multilevel mixed-effects models revealed no significant between-group differences at individual time points, although significant group-by-time interactions were observed (Table 2). The unadjusted model showed ORs increasing from 1.05 (95% CI, 0.60-1.84) at 12 months to 1.20 (95% CI, 0.68-2.11) at 24 months and 1.48 (95% CI, 0.84-2.61) at 36 months. This pattern of increasing effects persisted in model 1 and model 2. In model 2, ORs increased from 1.15 (95% CI, 0.78-1.70) at 24 months to 1.42 (95% CI, 0.96-2.11) at 36 months, with a significant group-by-time interaction (*P* = .04).

**Table 2.** Intervention Effects on HPV Vaccination Initiation and Completion by Study Year^a^

Time point	Odds ratio (95% CI)						
	HPV vaccination initiation			HPV vaccination completion		
	Unadjusted	Model 1^b^	Model 2^c^	Unadjusted	Model 1^b^	Model 2^c^
At 12 mo	1.05 (0.60-1.84)	1.04 (0.70-1.53)	1.03 (0.69-1.52)	1.17 (0.67-2.04)	1.18 (0.86-1.61)	1.06 (0.79-1.42)
At 24 mo	1.20 (0.68-2.11)	1.18 (0.80-1.74)	1.15 (0.78-1.70)	1.08 (0.62-1.88)	1.08 (0.79-1.47)	0.96 (0.72-1.29)
At 36 mo	1.48 (0.84-2.61)	1.46 (0.99-2.16)	1.42 (0.96-2.11)	1.39 (0.80-2.44)	1.41 (1.03-1.94)	1.24 (0.92-1.67)
*P* value for group-by-time interaction	.04	.03	.04	.04	.04	.05

Abbreviations: HPV, human papillomavirus; ICC, Intraclass correlation.

^a^ Results from mixed-effects logistic regression with practice and clinician as random effects. Results show intervention vs control at 12 months, 24 months (end of intervention), and 36 months (after intervention). Intention-to-treat analysis.

^b^ Adjusted for clinician baseline HPV vaccination rate. HPV vaccination initiation ICC = 0.03 for practice, ICC = 0.03 for clinician; HPV vaccination completion ICC = 0.02 for practice, ICC = 0.03 for clinician.

^c^ Additionally adjusted for patient sex, race, insurance, and clinician sex.

Similarly, for HPV vaccination completion by 13 years of age, the unadjusted model showed that intervention effects increased over time (12 months: OR, 1.17 [95% CI, 0.67-2.04]; 36 months: OR, 1.39 [95% CI, 0.80-2.44]; *P* = .04). In model 1, the comparison between groups showed no significant differences at 24 months (OR, 1.08 [95% CI, 0.79-1.47]), but significant differences emerged at 36 months (OR, 1.41 [95% CI, 1.03-1.94]), representing an 8.6 percentage point advantage for the intervention group (eTable 4 in Supplement 2). Model 2 showed a similar pattern of ORs (24 months: OR, 0.96 [95% CI, 0.72-1.29]; 36 months: OR, 1.24 [95% CI, 0.92-1.67]); however, the difference at 36 months was no longer significant, and the increasing pattern did not reach significance (*P* = .05).

Insurance type significantly moderated the intervention effect on HPV vaccination completion, yet no single insurance-specific effect reached statistical significance. Intervention effects did not differ by patient sex or race or by clinician’s sex (eFigure 2 in Supplement 2).

### Clinician Knowledge and Practice

Clinician survey response rates were 82.6% (38 of 46) in the intervention group vs 80.0% (32 of 40) in the control group at baseline, 84.1% (37 of 44) in the intervention group vs 59.0% (23 of 39) in the control group at 12 months, and 83.7% (36 of 43) in the intervention group vs 68.4% (26 of 38) in the control group at 24 months. Male clinicians and more experienced clinicians in the intervention group were less likely to respond; therefore, models were adjusted for clinician sex and years in practice (eTable 5 in Supplement 2).

The intervention had consistent effects on clinician communication styles and self-efficacy. Overall, clinicians in the intervention group reported more frequent announcement-style communications (difference, 0.70 [95% CI, 0.02-1.38]; *P* = .04) and a higher self-efficacy in addressing parental vaccine hesitancy (difference, 0.71 [95% CI, 0.05-1.36]; *P* = .04). These effects were significant at 12 months for both announcement-style communication (difference, 0.82 [95% CI, 0.04-1.60]) and self-efficacy (difference, 0.84 [95% CI, 0.04-1.65]). At 24 months, effects were attenuated and no longer statistically significant (announcement-style difference, 0.58 [95% CI, −0.05 to 1.50]; self-efficacy difference, 0.57 [95% CI, −0.22 to 1.36]).

## Discussion

This multicomponent intervention aimed at increasing HPV vaccination rates by 13 years of age demonstrated no significant between-group differences at any individual time points, although a pattern of increasing effects over time was observed for vaccine initiation. Vaccination rates were consistently higher in intervention practices at each interim assessment, with effect magnitude growing progressively. While the intervention potentially influenced vaccination behaviors during implementation, the effect magnitude was insufficient to achieve statistical significance; potential contributing factors included limited statistical power, pandemic disruptions, and practice variability. The growth of these differences, captured by the significant group-by-time interaction, indicates a cumulative intervention effect whereby early improvements in clinician communication, self-efficacy, and practice-level systems likely contributed to the increasing effects observed across time points. This gradual increase in effect magnitude suggests that this multicomponent strategy, designed to build sustainable systems for delivering evidence-based preventive care, required time to translate into measurable increases in vaccination rates.

The COVID-19 pandemic significantly affected our intervention’s timing (Figure 2). During the initial intervention (2020-2021), practice facilitators adapted their approach to support immediate practice needs, addressing clinical staff concerns, workflow adaptations, and pandemic-related disruptions. Although essential for maintaining practice engagement and building trust, this adaptation delayed the full implementation of HPV vaccination strategies, creating an unanticipated lag between clinician skill development and measurable practice change.

The intervention improved clinician announcement-style communication skills and self-efficacy as early as 12 months. However, translating these clinician-level improvements into increased vaccination rates requires time for practice-level integration,^42^ complicated by pandemic disruptions to health care delivery.^43^ Although clinicians gained skills through training, application was compromised by practice disruptions, competing priorities, and staff turnover.^19,44^ As pandemic pressures subsided and practice operations stabilized, clinicians could more effectively implement acquired skills, potentially explaining the largest effect magnitude observed at 36 months.

Multicomponent interventions have demonstrated effectiveness for HPV vaccination.^45,46^ Prepandemic studies, including a study by Perkins et al,^25^ showed sustained effects after the intervention. However, pandemic-era studies revealed challenges from increased vaccine hesitancy and health care system disruptions.^43,47^ Plate et al^48^ found that clinician attitude changes did not immediately translate into increased HPV vaccination rates during or after the intervention. Similarly, Szilagyi et al^27^ noted that their recent bundled intervention achieved effects similar to those seen with prepandemic communication strategies training alone.^24,30^ Our findings extend this literature by demonstrating an alternative pattern for HPV vaccination intervention: effects may be modest during intervention, particularly under challenging conditions, but grow over time as practices internalize new systems.

Our findings have implications for designing and evaluating practice-based HPV vaccination interventions. First, practices should anticipate that the benefits of systems-focused interventions may require 12 or more months after implementation activities conclude, and the evaluation timelines should be calibrated accordingly. Second, improvements in clinician communication and self-efficacy may serve as leading indicators of vaccination rate changes, although the association between these intermediate outcomes and vaccination rates may be influenced by the practice or external health system factors. Future studies should examine optimal intervention duration and adaptive implementation strategies for complex practice change, particularly as health care systems continue to face evolving challenges beyond COVID-19.^49^

### Limitations

This study has some limitations. Although the cluster randomized design with 20 practices provided appropriate practice-level randomization, it reduced statistical precision, contributing to wider 95% CIs at each time point. The small cluster number allows chance baseline imbalances, which we addressed through covariate adjustment. Despite comparable baseline HPV vaccination rates, unmeasured confounders related to social inequalities may have influenced the results. Future studies could stratify randomization on key variables proxying underlying disparities.

Second, the multicomponent intervention precluded isolating the effectiveness of individual components. We could not quantify the specific contributions of practice-level system changes, clinician communication training, or self-efficacy improvement to the observed vaccination rates, nor could we test other potential mechanisms of action. Therefore, we cannot determine which individual or combined components contributed to the observed increase in the intervention at different time points. In addition, although practice facilitation followed a standardized framework, delivery was tailored to each practice’s priorities, and delivery shifted largely to virtual facilitation due to the pandemic. These adaptations limited our ability to attribute outcomes to a uniform implementation or to compare the effectiveness of virtual vs in-person facilitation.

Furthermore, the baseline HPV vaccination rate (70.4%) exceeded Missouri’s mean vaccination rate (57%),^50^ potentially limiting observable improvement. Although insurance type moderated intervention effectiveness, no within differences were found; underlying factors require further investigation. Missing race data and subsequent imputation may have introduced bias. Differential clinician survey response rates between groups may have resulted in nonresponse bias. Finally, findings from a single geographic area may not generalize to other settings, and we could not objectively measure the effects of the pandemic.

## Conclusions

In this cluster randomized clinical trial of 20 pediatric practices, the multicomponent intervention did not significantly increase HPV vaccination rates by 13 years of age at individual time points. However, intervention effects significantly increased over time on initiation, suggesting the system-focused interventions may require extended evaluation periods to demonstrate measurable improvements.

## Notes

Supplement 1.
Trial Protocol

Supplement 2. **eTable 1.** Examples of Practice Facilitation Support **eTable 2.** Suggested Responses to Parent/Caregiver Questions **eFigure 1.** Key Elements of Communication Strategy **eTable 3.** Number of Clinicians and Patients by Practice at 12, 24, and 36 Months **eTable 4.** Intervention Effects on HPV Vaccination Initiation and Completion by Study Year: Percentage Point Differences **eFigure 2.** HPV Vaccination Initiation and Completion by Patient and Clinician Characteristics **eTable 5.** Clinician Characteristics Between Responders and Nonresponders Within Each Group
eReferences.

Supplement 3.
Data Sharing Statement
