# Supplementary material for: A Multicomponent Strategy to Increase Human Papillomavirus Vaccination Rates in Primary Care: A Cluster Randomized Clinical Trial
Source: JAMA Netw Open. 2026 Feb 26;9(2):e260049. doi: 10.1001/jamanetworkopen.2026.0049 (PMC12947024; doi:10.1001/jamanetworkopen.2026.0049)
Supplement: Supplement 1. — Trial Protocol [file jamanetwopen-e260049-s001.pdf]

Randomized Controlled Trial to Evaluate an Implementation Strategy to Increase Optimal Use of HPV Vaccine in Primary Care

National Clinical Trial (NCT) Identified Number: NCT04180462.

Principal Investigator: Allison King

Sponsor: Washington University School of Medicine

Grant Title: Randomized Controlled Trial to Evaluate an Implementation Strategy to Increase Optimal Use of HPV Vaccine in Primary Care

Grant Number: R01CA2356515-01A1

Funded by: National Institute of Health

Version Number: 1.12

29 April 2025

Summary of Changes from Previous Version:

Affected Section(s)	Summary of Revisions Made	Rationale
1.3, 8.1	Added a provider survey to assess implementation outcomes including acceptability, appropriateness, and sustainability.	To assess implementation outcomes of the intervention, a provider survey will be completed to inform the acceptability, appropriateness, and sustainability.

Table of Contents

Statement of Compliance....3
Investigator's Signature....3
1 Protocol Summary....4
1.1 Synopsis....4
1.2 Schema....6
1.3 Schedule of Activities....6
2 Introduction....8
2.1 Study Rationale....8
2.2 Background....8
2.3 Risk/Benefit Assessment....12
2.3.1 Known Potential Risks....12
2.3.2 Known Potential Benefits....12
2.3.3 Assessment of Potential Risks and Benefits....13
3 Objectives and Endpoints....14
4 Study Design....16
4.1 Overall Design....16
4.2 Scientific Rationale for Study Design....16
4.3 Justification for Intervention....17
4.4 End-of-Study Definition....17
5 Study Population....18
5.1 Inclusion Criteria....18
5.2 Exclusion Criteria....18
5.3 Lifestyle Considerations....18
5.4 Screen Failures....19
5.5 Strategies for Recruitment and Retention....19
6 Study Intervention(s) or Experimental Manipulation(s)....21
6.1 Study Intervention(s) or Experimental Manipulation(s) Administration....21
6.1.1 Study Intervention or Experimental Manipulation Description....21
6.1.2 Administration and/or Dosing....21
6.2 Fidelity....24
6.2.1 INTERVENTIONIST TRAINING AND TRACKING....24
6.3 Measures to Minimize Bias: Randomization and Blinding....27
6.4 Study Intervention/Experimental Manipulation Adherence....27
7 Study Intervention/Experimental Manipulation Discontinuation and Participant Discontinuation/Withdrawal....29
7.1 Discontinuation of Study Intervention/Experimental Manipulation....29
7.2 Participant Discontinuation/Withdrawal from the Study....29
7.3 Lost to Follow-Up....29
8 Study Assessments and Procedures....30
8.3 Adverse Events and Serious Adverse Events....31
8.3.1 Definition of Adverse Events....31

8.4 Unanticipated Problems....31
8.4.1 Definition of Unanticipated Problems....31
8.4.2 Unanticipated Problems Reporting....31
9 Statistical Considerations....32
9.1 Statistical Hypotheses....32
9.2 Sample Size Determinations....33
9.3 Populations for Analyses....33
9.4 Statistical Analyses....33
9.4.1 General Approach....33
9.4.2 Analysis of the Primary Endpoint(s)....34
9.4.3 Analysis of the Secondary Endpoint(s)....35
9.4.4 Safety Analyses....36
9.4.5 Baseline Descriptive Statistics....36
9.4.7 Sub-Group Analyses....36
9.4.8 Tabulation of Individual Participant Data....37
9.4.9 Exploratory Analyses....37
10 Supporting Documentation and Operational Considerations....38
10.1 Regulatory, Ethical, and Study Oversight Considerations....38
10.1.2 Study Discontinuation and Closure....38
10.1.6 Safety Oversight....38
10.3 Abbreviations and Special Terms....39
10.4 Protocol Amendment History....41
11 References....43

STATEMENT OF COMPLIANCE

1. The trial will be carried out in accordance with International Council on Harmonisation Good Clinical Practice (ICH GCP) and the following:

   - United States (US) Code of Federal Regulations (CFR) applicable to clinical studies (45 CFR Part 46, 21 CFR Part 50, 21 CFR Part 56, 21 CFR Part 312, and/or 21 CFR Part 812).

   National Institutes of Health (NIH)-funded investigators and clinical trial site staff who are responsible for the conduct, management, or oversight of NIH-funded clinical trials have completed Human Subjects Protection and ICH GCP Training.

   The protocol, informed consent form(s), recruitment materials, and all participant materials will be submitted to the IRB for review and approval. Approval of both the protocol and the consent form(s) must be obtained before any participant is consented. Any amendment to the protocol will require review and approval by the IRB before the changes are implemented to the study. All changes to the consent form(s) will be IRB approved; a determination will be made regarding whether a new consent needs to be obtained from participants who provided consent, using a previously approved consent form.

INVESTIGATOR'S SIGNATURE

Principal Investigator or Clinical Site Investigator:

Signed: Date: 8/8/2024

Name*: Allison King
Title*: Professor of Pediatrics
Pediatrics - Hematology Oncology

Investigator Contact Information:

Affiliation*: Washington University
Address: 660 South Euclid Saint Louis, MO 63110
Telephone: 3144544291
Email: king_a@wustl.edu

1 PROTOCOL SUMMARY

1.1 SYNOPSIS

**Title:**	Randomized Controlled Trial to Evaluate an Implementation Strategy to Increase Optimal Use of HPV Vaccine in Primary Care	
**Grant Number:**	R01CA2356515-01A1	
**Study Description:**	We will complete a cluster-randomized trial to evaluate the effectiveness of a multi-component implementation strategy (the intervention) to increase use of the HPV vaccine according to CDC guidelines. Twenty community pediatric practices will be randomly assigned to the intervention or wait-list control condition.	
**Objectives*:**	Primary Objective:	Compare, at 24 months, the proportion of eligible preteens who receive 1st dose (primary endpoint) and 2nd dose (secondary endpoint) of HPV vaccine before their 13th birthday in practices that receive the intervention with practices in the wait-list control group.
	Secondary Objectives:	Compare these outcomes at 36 months to assess the sustainability of intervention benefit.<br><br>Determine whether the intervention effect on vaccination outcomes is mediated by providers' increased belief in their capabilities to provide on-time HPV vaccination (primary outcome) and by their increased use of a presumptive, announcement approach to recommend HPV vaccine (secondary outcome)<br><br>Explore if the intervention effect on vaccination outcomes is mediated by the degree of practice change as measured on an 18-item HPV vaccine delivery score.<br>Assess the implementation fidelity, the acceptability and appropriateness of the intervention for providers, parental satisfaction with care, and cost.
**Endpoints*:**	Primary Endpoint:	The proportion of eligible preteens who receive the 1st dose of HPV vaccine before their 13th birthday at 24

		months post randomization.<br><br>The proportion of eligible preteens who receive the 2nd dose of HPV vaccine before their 13th birthday at 24 months post randomization.
	Secondary Endpoint:	The proportion of eligible preteens who receive the 1st dose of HPV vaccine before their 13th birthday at 36 months post randomization.<br><br>The proportion of eligible preteens who receive the 2nd dose of HPV vaccine before their 13th birthday at 36 months post randomization.
**Study Population:**	Approximately 120 Community based pediatric primary care providers in the St. Louis metropolitan area , who work in the 20 participating practices	
**Phase* or Stage:**	3 (Low-risk behavioral intervention)	
**Description of Sites/Facilities Enrolling Participants:**	The study will be conducted at Washington University	
**Description of Study Intervention/Experimental Manipulation:**	The theory-based, multi-component intervention includes: 1) an educational video; 2) audit and feedback of vaccine coverage; 3) a communication strategy to improve discussion of HPV vaccine; and 4) practice facilitation to support practice change. The intervention will be delivered through a series of brief practice visits with the facilitator that occur every 1-4 weeks over 2 years. All providers (intervention and control groups) will receive information provided by the CDC to assist motivated providers who want to improve use of HPV vaccine that are representative of “usual care.” These will include the current recommendations, background information and the CDC’s patient educational materials and website. All providers will also receive Baseline HPV vaccination rates.	
**Study Duration*:**	5 years	
**Participant Duration:**	3-5 years	

1.2 SCHEMA

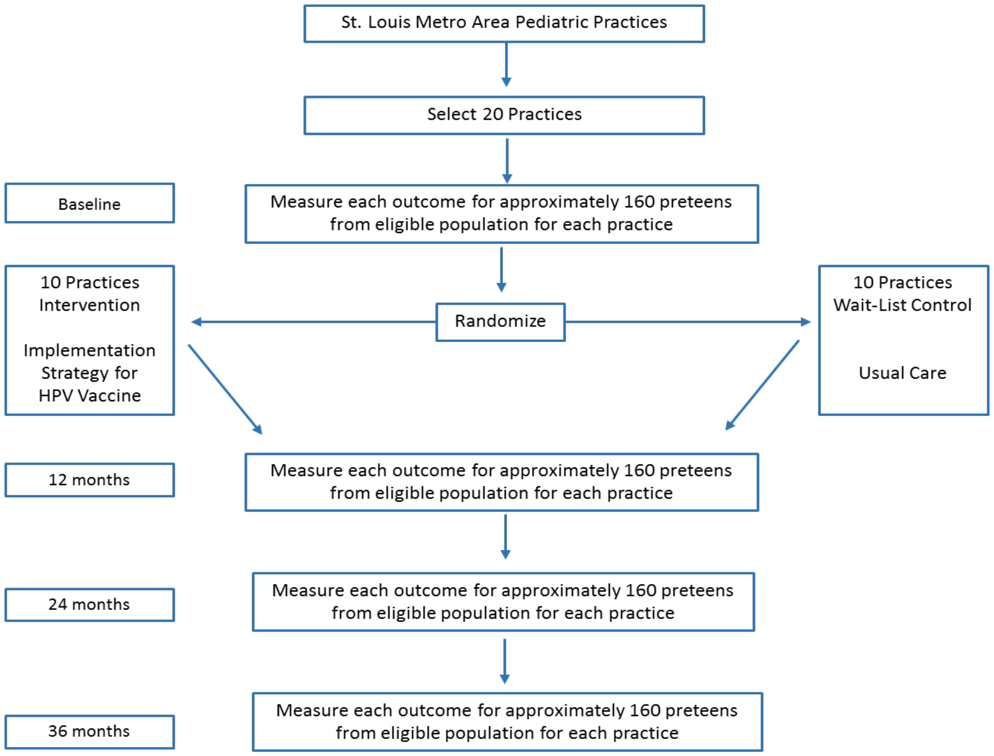

1.3 SCHEDULE OF ACTIVITIES

**Time**	**Participant**	**Study activities**
Baseline	Provider	Complete provider survey
	Practice	Complete review of EMR data
	Practice	Complete practice survey and assess HPV vaccine delivery
12-months	Provider	Complete provider survey
	Practice	Complete review of EMR data
	Practice	Assess HPV vaccine delivery
	Intervention Practice	Assess intervention fidelity

24-months	Provider	Complete provider survey
	Practice	Complete review of EMR data
	Practice	Assess HPV vaccine delivery
	Intervention Practice	Assess intervention fidelity
	Intervention Practice	Complete an exit interview
36-month	Practice	Complete review of EMR data
End of Study	Provider	Complete a survey to assess acceptability, appropriateness and sustainability of the intervention

2 INTRODUCTION

2.1 STUDY RATIONALE

The vaccine targeting oncogenic strains of the human papilloma virus (HPV) can prevent commonly occurring cancers if given to girls and boys before sexual debut. Yet, in the United States (U.S.), more than 10 years after introduction of the vaccine, fewer than half of the target population are vaccinated,[1,2] a far cry from the Healthy People 2020 goal of 80% coverage.[3] Even though vaccination coverage has been increasing among 13-17 year old females, 40% report receiving the vaccine after sexual debut, when vaccination benefit is less certain.[1] It is estimated that for every year of low vaccine coverage in the US, 4,400 girls will have a future cervical cancer.[4] Innovative strategies are urgently needed to increase optimal use of this effective, safe vaccine.

National recommendations from the Centers for Disease Control and Prevention (CDC) are to complete the 2-dose HPV vaccine series by age 13.[5] Uptake of the tetanus-diphtheria-pertussis booster (Tdap) and the meningococcal vaccine (MCV), two other vaccines required by age 13 for school attendance, are over 80% across the country.[2] Yet, vaccination of HPV vaccine remains low (~39% in 2017), with little year-to-year improvement in this age-group.[1,2] To date, efforts to increase vaccine use have, at best, increased vaccine initiation by 10% with no impact on vaccine completion by age 13 and no evidence of sustained benefit.

Our objective is to evaluate the effectiveness of a promising, innovative intervention to make significant improvements in optimal HPV vaccine use. The multi-component intervention is based on the Theoretical Domains Framework (TDF) theory of behavior-change and uses practice facilitation to support practice change. It includes strategies to increase provider communication skills and their belief in their capabilities to deliver HPV vaccine by age 13 and supports implementation of a comprehensive system for HPV vaccine delivery. Preliminary results from pilot testing in 6 practices show a 19-percentage point increase in HPV vaccine initiation in the 15-months from pre- to post-intervention (62% to 81%), reaching the target for Healthy People 2020.[3] A rigorous evaluation is needed to establish the effectiveness of the intervention in a generalizable sample prior to scaling for widespread use. In this study, the study population will comprise 20 primary care pediatric practices in the St. Louis metropolitan area , where only 40% of 13- 17 year-olds have completed the HPV vaccine series.[2] Vaccine use will be assessed at baseline, at 24 months to assess effectiveness of the intervention, and at 36 months to assess if change is sustained. We will investigate the mechanism of action of the intervention and assess features of the intervention that are important for scaling, including cost.

2.2 BACKGROUND

The full benefit to HPV vaccine to prevent cervical cancer and other genitourinary cancers is not being realized. HPV is the most common sexually transmitted virus in the U.S. with a prevalence of 33% among 14- to 19-year olds.[1] Several HPV strains are oncogenic causing 31,500 new urogenital and oral cancers in men and women each year.[6] Most of these cancers could be prevented by vaccination with HPV

vaccine prior to sexual debut as recommended by the CDC.[1,6] Yet, uptake of this cancer-preventing vaccine has been slow and in 2017, only 49% of adolescents ages 13 to 17 had completed the HPV vaccine series (53% of females, 44% of males) .[2] Self-reported data from the National Health and Nutrition Examination Survey (NHANES) suggest that although vaccination has significantly increased in the past 10 years among females in this age- group, 40% will not get the full benefit of the vaccine as they received the vaccine after initiating sexual activity.[1] Coverage by age 13 (i.e., before their 13th birthday), the age targeted by the CDC recommendations and a HEDIS quality measure, is lower for both girls and boys, although data for this age-group are not routinely reported. In 2017, uptake of HPV vaccine in 13-year-olds was 39% and previous studies have shown little year-to-year improvement in 11-12 year-olds.[1,6] There is an obvious disparity in uptake when compared with Tdap (88%) and MCV4 (82%), vaccines that are required by age 13 for school attendance.[6,7] It is estimated that for every year of low vaccine coverage, 4,400 girls will have a future cervical cancer.[4] Also, the annual direct cost of preventing and treating vaccine-preventable HPV-related disease is estimated to be $8.0 billion.[8] There is an urgent need for a large increase in the optimal use of this effective, safe vaccine.

To date, interventions to increase HPV vaccination have had limited success. Single-component interventions targeting parents and providers have had mixed results. Educational interventions targeting parents' belief that their child is not at risk for HPV infection have not been effective,[9] and parental hesitancy continues to be a barrier to vaccine use.[10,11] Provider-focused strategies such as reminder systems, audit and feedback, and educational outreach, while effective in increasing the use of other vaccines, have had inconsistent results for increasing HPV vaccination.[12 -14] When used concurrently, HPV vaccination increased by 5-10%,[12,13,15 -18] and in one study, improvements were sustained for at least six months.[19] Studies showing larger effects have been criticized for being methodologically deficient.[9,12] Policy change approaches such as requiring HPV vaccine for school attendance that have been effective in other countries have not been effective in the U.S., possibly due to generous opt-out provisions.[12,14] A systematic review of the literature indicated that high quality HPV vaccine communication was vital for increasing coverage,[20] yet few interventions have tried to improve provider communication skills. One study compared presumptive announcements, in which providers assume parent readiness to vaccinate, with conversational, explanatory approaches, and found presumptive announcements were more effective.[15] A recent randomized trial by Dempsey et al. showed that a 5-component communication intervention to support a presumptive recommendation for HPV increased initiation for 11-17 year-olds by 9%, with most improvement occurring at well-child visits.[21] Vaccine completion did not change in intervention sites and decreased in control sites. Sustainability of the impact of this intervention on vaccination rates was not assessed.

Primary care providers face considerable barriers when trying to implement the CDC recommendations for HPV vaccine.[22, 23] Over 60% of office-based primary care physicians, including pediatricians, work in practices with 5 or fewer physicians. [24] These practices are largely small-scale, independently run enterprises, operate on a narrow margin and lack the infrastructure to support practice change. [25 -28] Yet, a body of NIH-funded research suggests primary care practices are complex adaptive systems and interventions to effect sustained change in care delivery must themselves be sustained (~2years) and

tailored to the unique values, structures and processes for each practice. [28 -32] External motivators for improvement can be important.[28, 33, 34] Our formative work suggests two important external motivators that could encourage increased optimal use of the HPV vaccine: the requirement for active participation in an approved quality improvement (QI) activity for The American Board of Pediatrics (ABP) maintenance of certification (MOC); and financial incentives for “meaningful use of data” for practices designated as a patient-centered medical home. However, even those who are motivated to address this gap in care likely need help to change their clinical practice.

Practice facilitators support change in primary care practices by empowering and supporting providers and staff to engage in the change process together. Using participatory methods, practice facilitators help demystify improvement methods and support data-driven decision-making, tailoring their approach to meet practice needs. External facilitation has been used successfully in a wide range of primary care practice settings to increase preventive care for adults and children.[29, 35-41] Improvement in the desired clinical care activity varied by activity, but was consistently at least 10%. [36-38, 40, 41] Follow-up studies have demonstrated changes in care delivery were sustained for up to six years, suggesting they had become instutionalized.[29, 35] We did not identify any studies where practice facilitation was used to increase vaccination, but in one project to improve diabetes care, annual influenza vaccine increased by 15%.[32]

There is a critical need for theory-based implementation interventions to improve the use of HPV vaccine and sustain benefit. Improving use of evidence-based practices requires behavior change, and in order to design effective interventions and allow purposeful improvements when interventions fail, it is necessary to understand these behaviors in context. Models and theories of behavior and behavior change can be used to determine who and what needs to change and to identify behavior change strategies. To this end, Damschroder and colleagues developed the Consolidated Framework for Implementation Research (CFIR) to consolidate and unify key constructs from 19 published implementation theories.[42] The CFIR identifies five major domains that influence successful implementation: characteristics of the intervention, the inner setting (the context through which implementation will proceed), the outer setting (the context in which the organization resides), implementer’s characteristics, and the processes of implementation.[42] Michie and colleagues consolidated 33 theories of behavior and behavior change in order to develop a theoretical framework for understanding behavior change among health professionals.[43-45] Using consensus among experts, they identified a framework with 14 theoretical domains covering the main factors influencing provider’s clinical behaviors and behavior change (Theoretical Domain Framework, TDF).[43, 45,46] We have used the CFIR and TDF to inform development of our intervention as indicated in Figure 1.

Figure 1. Steps for Theory-based development of Implementation Intervention

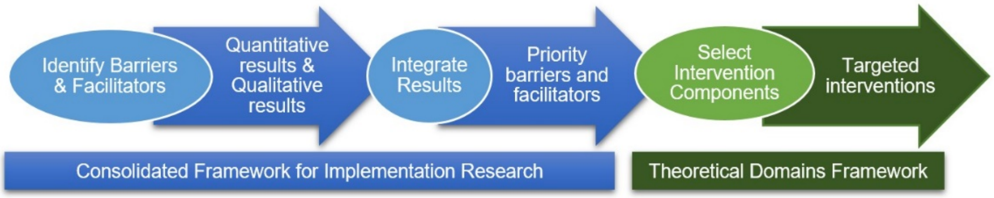

Guided by the CFIR,[42] we conducted semi-structured interviews with 16 providers from 10 practices to systematically identify barriers to HPV vaccination in pediatric practices [47] This investigation revealed that all providers were aware of the CDC recommendations for HPV vaccination and universally assumed responsibility for vaccine delivery. Typically, for both girls and boys, providers recommended the HPV vaccine at the 11-12-year-old check-up visit. All but one provider used an explanatory style for this discussion, and the strength of their recommendation varied. All reported that persuading hesitant parents to vaccinate was difficult and time consuming; some delayed these discussions until the child was older and the parents were more accepting. Teamwork to deliver the vaccine series was uncommon, and no providers were aware of their partners' approach to HPV vaccination or monitored vaccine coverage.

Using 2014 data, we found that vaccine initiation and completion by age 13 varied among providers from 2% to 26%, and 1% to 26%, respectively, and varied 4 to 5-fold among providers within the same practice. These data demonstrate the urgent need for changes in vaccine delivery in community-based practices in our community to increase optimal use of this vaccine. By comparing factors between providers with higher and lower HPV vaccine coverage, we identified [14] CFIR constructs across all five domains of the conceptual framework that were important barriers to following CDC guidelines.[48] Most distinguishing factors related to provider characteristics – their lack of buy-in to vaccination by age 13, their lack of confidence to address parental hesitancy, and poor communication skills to promote timely HPV vaccine use. Coverage was higher in practices with coordination between the provider and staff to ensure opportunities for vaccination were not missed. As described in our recent publication, [47] we crafted an implementation strategy (the intervention) to address these factors, guided by the TDF.[43, 46] The intervention was further developed during a practice quality improvement project, described below.

For ~15-months, a practice facilitator has worked with 6 practices (20 providers) to implement the intervention, first targeting initiation of HPV vaccine by age 13. All practices were provided with their baseline data and received education about the potential benefit for patients and the practice for following CDC recommendations, a communication strategy, and patient education materials. They identified a QI team and worked with the facilitator towards the practice's goals for improvement. Each practice completed Plan-Do-Study-Act (PDSA) cycles, testing different strategies to improve care. PDSA cycles are small cycles of change that involve reviewing data to assess gaps in performance, identifying possible changes to improve care, selecting and testing an improvement strategy, and monitoring and adjusting new processes for care delivery. To date, the number of team meetings with the facilitator varied from 7 to 11, 10% were by phone, and each visit lasted < 1 hour.

For each provider, HPV initiation was assessed on a randomly selected sample of ~30 patients, 11.0 to 13.0 years old with at least one practice visit (well-child or acute care visit) between June 1st and September 30th in the measurement period. These included 2016, 2017, and 2018. Overall, there was a 19-percentage point increase in HPV vaccine initiation in the 15- months from pre- to post-intervention (62% to 81%), reaching the target for Healthy People 2020.[3] Results varied within and across practices as shown in Figure 2.

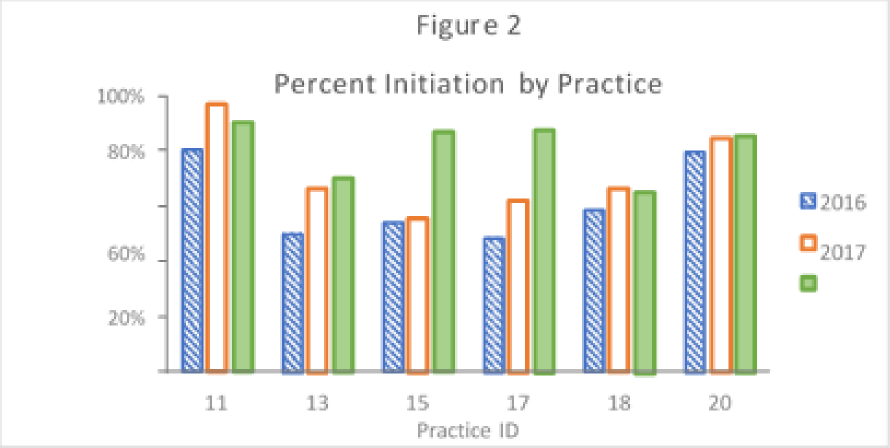

Figure 2

Percent Initiation by Practice

Efforts to impact vaccine completion by age 13 were initiated in March 2018, and after only 7 months, we observed a 12% increase. The majority (>80%) of providers felt the following features of the intervention were very important in the change process: access to the practice facilitator, the QI practice team, help with measurement and review of vaccination data, and the communication strategy, and all would strongly recommend the program to their colleagues

These findings suggest that the proposed 2-year intervention is feasible and likely to have a large impact and sustained benefits.

2.3 RISK/BENEFIT ASSESSMENT

2.3.1 KNOWN POTENTIAL RISKS

There are no immediate or long term physical or psychological risks associated with study participation. There are no study products. The intervention promotes national recommendations for use of HPV vaccine, a safe, effective vaccine for cancer prevention.

There is a possibility that during routine chart audits, confidentiality could be compromised.

2.3.2 KNOWN POTENTIAL BENEFITS

A long-term benefit to society could be the identification of an effective intervention to increase optimal use of the cancer preventing HPV vaccine. Immediate and long-term benefits for participating practices and providers include development of effective and efficient vaccine delivery systems and increased capability to strongly recommend the HPV vaccine and address the concerns of hesitant parents. Also, their experience in the change process may encourage them to identify and address other care gaps in

their practice. An immediate benefit for preteens attending study practices could be receipt of HPV vaccine before sexual debut, allowing maximum benefit from this safe vaccine.

2.3.3 ASSESSMENT OF POTENTIAL RISKS AND BENEFITS

There are essentially no significant immediate or long-term risks in study participation. The potential benefits to society and children from an effective intervention to reduce HPV-related cancers far outweigh the potential risks.

3 OBJECTIVES AND ENDPOINTS

OBJECTIVES	ENDPOINTS	JUSTIFICATION FOR ENDPOINTS	PUTATIVE MECHANISMS OF ACTION
Primary			
To evaluate the effectiveness of the intervention to improve optimal HPV vaccine use.	The proportion of eligible preteens who initiate the HPV vaccine before their 13th birthday, at 24 months.<br><br>The proportion of eligible preteens who complete the HPV vaccine before their 13th birthday, at 24 months.	Consistent with CDC Guideline Recommendations	
Secondary			
To assess the sustainability of intervention benefit.	The proportion of eligible preteens who initiate the HPV vaccine before their 13th birthday, at 36 months.<br><br>The proportion of eligible preteens who complete the HPV vaccine before their 13th birthday, at 36 months.		
Tertiary/Exploratory			
To determine whether the intervention effect on vaccination outcomes is mediated by providers' increased belief in their capabilities to provide on-time HPV vaccination (primary outcome) and by their increased use of a presumptive, announcement approach to recommend HPV vaccine (secondary)	The proportion of eligible preteens who initiate the HPV vaccine before their 13th birthday, at 24 months.<br><br>The proportion of eligible preteens who complete the HPV vaccine before their 13th birthday, at 24 months.		Mediation

OBJECTIVES	ENDPOINTS	JUSTIFICATION FOR ENDPOINTS	PUTATIVE MECHANISMS OF ACTION
To explore if the intervention effect on vaccination outcomes is mediated by the degree of practice change as measured on an 18-item HPV vaccine delivery score.<br><br>To inform scalability of the intervention, we will assess implementation fidelity, the acceptability and appropriateness of the intervention for providers, parental satisfaction with care, and cost.	The proportion of eligible preteens who initiate the HPV vaccine before their 13th birthday, at 24 months.<br><br>The proportion of eligible preteens who complete the HPV vaccine before their 13th birthday, at 24 months.<br><br>Implementation fidelity, the acceptability and appropriateness of the intervention for providers, parental satisfaction with care, and cost.		Mediation

4 STUDY DESIGN

4.1 OVERALL DESIGN

We will complete a single site, cluster-randomized trial to evaluate the effectiveness of a multi-component implementation strategy (the intervention) to increase use of the HPV vaccine according to CDC guidelines. Randomization will occur after baseline assessment and the unit of randomization is a community-based, primary care pediatric practice. The randomization schema will be generated and implemented by the study statistician. Twenty practices will be randomized into two groups, the 2-year intervention group (n=10) or a wait-list control group (n=10). The intervention includes: 1) an educational video to increase the provider's knowledge about guideline recommendations and patient and practice benefits of vaccination by age 13; 2) audit and feedback of vaccine coverage to increase motivation to engage in practice change; 3) a communication strategy to improve the provider's communication skills and their self-efficacy to address parental hesitation; and 4) practice facilitation to support practice change to develop a sustainable HPV vaccine delivery system. The intervention will be delivered through a series of brief practice visits with the facilitator that occur every 1-4 weeks over 2 years. Vaccine use will be assessed at the practice level using 12-months of routinely collected clinical data to allow for seasonal variation in care delivery patterns. Vaccine use will be assessed at baseline, at 12 and 24 months to assess effectiveness, and at 36 months to assess if change is sustained. Due to the 2020 pandemic and overall concerns for safety, we will use ZOOM video conferencing for research activities, including obtaining measures and implantation of the intervention

The study hypotheses are: 1) *The 2-year intervention will increase the percentage of preteens who receive HPV vaccine before their 13th birthday by at least 13 percentage points, and benefits will be sustained at 36 months. 2) The intervention will increase on-time HPV vaccination by increasing providers' capability (communication skills) and their motivation (confidence in their capabilities) to deliver on-time HPV vaccination*.

4.2 SCIENTIFIC RATIONALE FOR STUDY DESIGN

A rigorous evaluation such as the proposed cluster randomized controlled trial is needed to establish the effectiveness of the intervention in a generalizable sample prior to scaling for widespread use. Randomization will be at the level of the practice for two reasons: 1) the intervention is designed to change the system for adolescent vaccine delivery at the practice level, and 2) to prevent contamination of provider behavior within the same practice. The 3-year follow-up required for assessment of sustained change, a key consideration prior to widespread dissemination, precluded a stepped wedge design.

Practices will be recruited from community pediatric practices in the St. Louis metropolitan area and are representative of independent practices where vaccination typically occurs. Our sample size of 20 practices (~80 providers) allows us to test the primary hypothesis and provide interpretable results. All providers (intervention and control groups) will receive information provided by the CDC to assist motivated providers who want to improve use of HPV vaccine that are representative of "usual care." These will include the current recommendations, background information and the CDC's patient educational materials and website. The wait-list control group will be offered the intervention after the evaluative study is completed. Initiation of HPV vaccine by age 13 is the primary outcome as local providers suggested this is the most difficult barrier to overcome to achieve optimal use of this effective vaccine. Vaccine outcomes will be assessed at the clinic level using 12-months of routinely collected clinical data to allow for seasonal variation in care delivery patterns (the majority of check-up visits occur in the summer). This measurement approach is characteristic of quality metrics and is needed to show change in vaccine coverage over time, as the guidelines are age-specific. The outcome assessor will be blinded to study group allocation to minimize measurement bias.

4.3 JUSTIFICATION FOR INTERVENTION

The reasons for under-use and sub-optimal use of HPV vaccine are complex and require an innovative solution. A systematic assessment of the HPV vaccination process identified that the barriers to use were related to provider and practice characteristics. We developed a multi-component intervention designed to simultaneously address all these factors. Previous research has shown that interventions to change physician behavior are most effective if they are based on national guidelines, address specific barriers to change, and include several components.[49,50] The proposed intervention is based on CDC guidelines, addresses specific barriers to HPV vaccine use in primary care practices, and has multiple components. It is informed by the Theoretical Domain Framework, a behavioral change theory specific for primary care providers, and has been refined by pilot testing. It is designed for sustainability, using practice facilitation to support practice change, allowing 2 years for sustained change to occur. Preliminary testing suggest the intervention is effective, acceptable to providers and ready for community-level testing.

4.4 END-OF-STUDY DEFINITION

Intervention practices are considered to have completed the study after the baseline, 12-, 24- and 36-month measurements for vaccine coverage have been collected. Control practices may continue for an additional 24 months to receive the intervention.

5 STUDY POPULATION

5.1 INCLUSION CRITERIA

IN ADDITION TO PROVIDING APPROPRIATE CONSENT, PRACTICES AND INDIVIDUALS MUST MEET THE FOLLOWING INCLUSION CRITERIA TO PARTICIPATE IN THE STUDY. FOR EACH GROUP OF STUDY SUBJECTS, PARTICIPATION IS OPEN TO ALL, INCLUDING ANY GENDER AND RACIAL AND ETHNIC GROUP.

**Practice**	**Providers**		**Adolescents**
Community-based pediatric primary care practices with 2 or more providers	Providers who deliver wellness care to preteens and adolescents		Adolescents 10-13 years of age during the measurement period
Practice in St. Louis metropolitan area	Willingness to complete study questionnaires		At least one visit during the measurement period
Willingness to participate in the practice facilitator-guided QI process	Willingness to participate in the practice facilitator-guided QI process		

5.2 EXCLUSION CRITERIA

Practices, providers and parent/caregivers may be excluded at the discretion of the PI. Our plan is to exclude practices that are solo-practitioners and that have paper records. If we have difficulty recruiting, we may include these practices.

5.3 LIFESTYLE CONSIDERATIONS

N/A

5.4 SCREEN FAILURES

N/A

5.5 STRATEGIES FOR RECRUITMENT AND RETENTION

Practices: We will invite all potentially eligible practices that are affiliated with WU PAARC and Community Outpatient Practice Experience (COPE) to participate. The first 20 practices that agree to participate will be randomly assigned to the intervention or control group using an allocation ratio 1:1.

Practices may be contacted by email, fax, letter, phone, or in-person to invite participation. All providers in the practice will be invited to participate. Dr. Garbutt and/ or a member of the study team will meet with those who are interested in learning more about the study to explain the details of study participation and answer any questions. These meetings will be held at the provider's office. If we experience difficulty with recruitment, we may expand our criteria to include solo practitioners and practices who still maintain paper records, as well as practices in the community that are not affiliated with WU PAARC or COPE.

We anticipate that we will recruit ~80 primary care pediatricians from the 20 participating practices. We estimate the racial/ethnic breakdown of participating physicians based on surveys completed by members of our PBRN, the available study population. We estimate that 27% of participating physicians will be male, ~84% will be Caucasian, 6% African American, 7% Asian. About 6% will be Hispanic.
Due to the 2020 Coronavirus pandemic, the baseline parent survey was eliminated. Changes in EMR protocols has made parent contact information inaccessible, therefore the electronic parent survey has been completely eliminated from the study. The goal of this survey, to assess if parental satisfaction with HPV vaccine care differed between study groups, will be met by adding questions to the provider survey. Specifically, to assess provider experiences with patient complaints and loss to the practice related to HPV vaccine delivery, repeating the measure from the baseline provider survey.

Incentives: We will provide financial compensation for time taken to participate in the study including $500/year for the business entity of each participating practice for administrative expenses (obtaining the lists of eligible patients, allowing study team access to medical records, etc.). We will also provide $50 for providers who complete study surveys.

We will provide opportunity for all participating pediatricians to earn American Board of Pediatrics (ABP) Maintenance of Certification (MOC) Part-4 credit to maintain board certification in general pediatrics. To accomplish this, pediatricians must be up to date in four areas including professional standing (part 1), lifelong learning (part 2), cognitive expertise (part 3) and improving professional practice (part 4). Every 5 years, each pediatrician is required to earn 100 points through part 2 and part 4 activities, with 40-60 points for each part. Part 4 activities require pediatricians to demonstrate competence in systematic measurement and improvement in patient care work that is intended to improve child health. Our

project is approved by the American Board of Pediatrics and is sponsored by Saint Louis Children's Hospital, Washington University School of Medicine, and meets requirements for part 4 activities. To earn MOC-credit, the pediatrician must participate in the intervention for at least 12-months.

We will consult with our 6-member Advisory Board of key stakeholders (pediatricians, pediatric nurse practitioners and parents) to receive their advice about strategies to increase recruitment and retention of practices.

6 STUDY INTERVENTION(S) OR EXPERIMENTAL MANIPULATION(S)

6.1 STUDY INTERVENTION(S) OR EXPERIMENTAL MANIPULATION(S) ADMINISTRATION

6.1.1 STUDY INTERVENTION OR EXPERIMENTAL MANIPULATION DESCRIPTION

The intervention occurs at the level of the practice.

All providers (intervention and control groups) will receive information provided by the CDC to assist providers to improve use of HPV vaccine including the current recommendations, background information about the vaccine, and patient educational materials. The CDC website will be distributed. Baseline HPV vaccination rates will also be given to all providers.

The intervention:
The theory-based, multicomponent intervention includes: 1) an educational video to increase the provider's knowledge about guideline recommendations and patient and practice benefits of vaccination by age 13; 2) audit and feedback of vaccine coverage to increase motivation to engage in practice change; 3) a communication strategy to improve the provider's communication skills and their self-efficacy to address parental hesitation; and 4) practice facilitation to support practice change to develop a sustainable HPV vaccine delivery system. The intervention is based on CDC guidelines, addresses specific barriers to HPV vaccine use in primary care practices, and has multiple components. It is informed by the Theoretical Domain Framework, a behavioral change theory specific for primary care providers, and has been refined by pilot testing. We anticipate that the intervention will increase on-time HPV vaccination by increasing providers' capability (communication skills) and their motivation (confidence in their capabilities) to deliver on-time HPV vaccination.

The intervention is designed for sustainability, using practice facilitation to support lasting practice change. It will be delivered through a series of brief practice visits with the facilitator that occur every 1-4 weeks over 2 years. The usual care control group will be offered the intervention after the evaluative study is completed. Visits will be completed either in-person or through zoom video conferencing if face-face visits are not possible.

6.1.2 ADMINISTRATION AND/OR DOSING

Practice facilitation comprises on-site visits by the facilitator to meet with providers and/or staff. The schedule of visits is tailored to the practice but generally visits occur twice in the first month (to make a practice assessment) and then weekly to monthly thereafter (to effect practice change, provide education etc), with the goal of practice providers and staff working together to enhance their ability to deliver on-time HPV vaccination and encouraging persistence in the change process. Additional support via telephone and/or email will be available. Our own experience and reports in the literature[31, 63] support the need for an extended time period for the change process to occur, with multiple PDSA

cycles. Practices will need time to expand their clinical focus to a population-based approach to identify and vaccinate eligible preteens who do not attend for a check-up visit.[31] Strategies to identify and recall these patients will be new for many practices and include software, EMR features and staff outreach. In addition, completion of the vaccine series requires 6 to 12 months. Thus, practices will have access to the practice facilitator and intervention components for 2 years, including two "well-child" seasons, a time when providers in the pilot study reported it was easier to access the target age group and experiment with changes to HPV vaccine delivery.

Table 2. Roll-out of the intervention

**Month 1**	
**Visit**	**Activities**
Visit 1<br>Led by:<br>Practice Facilitator and Principal Investigator and/or Project Coordinator<br>(30-45 mins)	▪ Review project goals<br>▪ Identify overall practice goals<br>▪ Introduce intervention components<br>▪ Introduce practice facilitator and review facilitation process<br>▪ Identify potential members for practice QI team<br>▪ Introduce practice assessment process
Practice Assessment with practice providers, practice manager, and/or staff involved in vaccine process<br>Led by:<br>Practice Facilitator	▪ Understand provider and practice processes for giving HPV vaccine<br>▪ Understand practice workflow<br>▪ Learn individual provider goals for project<br>▪ Ask providers to participate in education by watching video
Visit 2<br>Led by:<br>Practice Facilitator<br>(30-45 mins)	▪ Review baseline HPV vaccination rates for practice/U.S.<br>▪ Synthesize current HPV vaccination practices, provider individual project goals, and practice goals<br>▪ Discuss potential opportunities for improvement<br>▪ Establish practice goals for initiation and completion<br>▪ Decide focus for initial change idea targeting initiation<br>▪ Identify resources needed to accommodate change

**Month 2**	
Visit 3<br>Led by:<br>Practice Facilitator<br>(30-45 mins)<br>(no more than 2-3 weeks after Visit 2)	▪ Determine first PDSA cycle to accomplish change idea<br>▪ Identify process needed to accommodate change<br>▪ Identify resources needed to accommodate change<br>▪ Decide how to monitor change
**Month 3 – 2 Years**	
Subsequent visits during change process<br>Bi-weekly/monthly visits for 2 years<br>(15-45 mins)	▪ Review success with PDSA cycle(s) and determine next steps<br>▪ Review considerations to implement change ideas<br>▪ Determine process for implementing change ideas and PDSA cycles<br>▪ Support practice QI team and efforts to make changes<br>▪ Provide education and skills training for providers and staff such as communication strategy, HPV vaccine, parental talking points, etc.<br>▪ Assist with self-monitoring of HPV vaccination to assess progress<br>▪ Assist with measurement for PDSA cycles<br>▪ Provide social support<br>▪ Share other primary care practices' best practices and change ideas<br>▪ Provide education for providers and staff about QI methods
Subsequent visits for Maintenance<br>Led by:<br>Practice Facilitator<br>(check in every 2 weeks to monthly for 3 months)	▪ Continue monitoring practice to check for sustainability<br>▪ Work with practice to trouble shoot any changes or procedures as needed<br>▪ Make sure the practice has access to all resources and tools as needed<br>▪ Provide process for obtaining MOC-Part 4 credits

6.2 FIDELITY

6.2.1 INTERVENTIONIST TRAINING AND TRACKING

Ms. Dodd is responsible for training the practice facilitators, and for their supervision throughout the study period. Training is based on the AHRQ Practice Facilitator training and modules, accepted by the University of Buffalo Practice Facilitation Program.

Fidelity criteria were developed by the study team based on a literature review, best practices for facilitation [71, 72] and our experiences using facilitation in the pilot study. Every 12-months, use of the core components will be assessed and recorded by Ms. Dodd using a 23-item observational measure (Table 3). All items will be scored on a 2-point scale (yes/no) or a percentage basis. [73] We will also track the time to achievement of key implementation milestones by noting dates of completion of specific activities (such as completion of provider education).[74] The facilitators will record the number and duration of visits to the practice, number of QI team meetings and attendees, and PDSA cycles undertaken. To supplement these measures, guide the intervention and assess the intensity of the facilitation process, the facilitator will keep detailed field notes about practice observations and interactions that will be reviewed with Ms. Dodd at weekly meetings.

Table 3. Fidelity Score

HPV Vaccine Project Fidelity Score	Adherence		Dose	
**Audit and Feedback**	**Yes**	**No**		
Baseline data summary report is available				
Facilitator provides practice data and de-identified provider-level data to the QI team.			QI team reviews practice baseline data.	Y/N
Facilitator provides identified individual data to each provider			Providers who review own baseline data.	%
Facilitator encourages reflection and critical assessment of current practice and identification of performance gap(s).				
**Provider/Staff education**	**Yes**	**No**		
Facilitator reviews guideline recommendation for "on time" HPV vaccine with QI team.			Providers participate in education/training about “on-time” HPV vaccination (video, other).	%
Facilitator reviews rationale for age target with QI team.				

Facilitator provides educational resources to practices including guideline recommendations, videos, manuscripts.			HPV vaccine training provided to staff.	Y/N
**Communication Strategy**	**Yes**	**No**		
Facilitator educates providers and staff about the four key elements of the communication strategy.			Providers and key staff receive education re 4 key elements.	P= %<br>S= %
Facilitator provides communication training opportunities for providers and staff.			Providers and key staff participate in communication strategy training.	P= %<br>X RN & X PM
			Number of PDSA cycles targeting communication strategy.	#
**Practice Facilitator to support using QI methods to effect change**	**Yes**	**No**		
Facilitator meets individually with providers and practice manager.			Providers who meet with PF for baseline assessment.	%
			Meet with practice manager and/or staff for baseline assessment.	Y/N
Facilitator completes flow diagram summarizing current care, integrating baseline assessment and chart review data.				
Facilitator presents flow diagram summary to QI team.			QI team members who review flow diagram summary.	%

Core QI team comprises providers and staff.			Providers on initial QI team.	%
**HPV Vaccine Project Fidelity Score**	**Adherence**		**Dose**	
**PF to support using QI methods to effect change (cont.)**	**Yes**	**No**		
Additional staff included as needed			QI team members who remain on team for 1 year.	%
Number of QI team meetings in year 1 (count in-person visits, virtual meetings).			Total time spent (hours) in QI team meetings for providers, staff, and PF.	P=<br>S=<br>PF=
Number of additional PF contact with practice/year.				
QI team address at least 2 change ideas per year to meet their targets for 'on time' delivery of HPV vaccine.			Number of changes accomplished for initiation and completion (determined by QI team).	I=<br>C=
QI team identifies metrics to monitor.			Number of completed measurement reviews of HPV vaccine use/year.	
QI team implements system to monitor.				

Practice Facilitator

**Quality of delivery**: score 1 (poor) to 10 (excellent)	**Score**
PF ability to identify, assess, and summarize relevant data - consider baseline assessment, PDSA cycle measures and on-going monitoring	
PF ability to communicate effectively with QI team	

PF ability to encourage interactive problem solving and consensus building	
PF ability to encourage a diverse QI team including key personnel	
PF ability to encourage and sustain change efforts eg, shares info across practices, celebrates etc.	
PF ability to tailor facilitation activities to practice needs and circumstances	

Overall

**Quality of delivery**: score 1 (poor) to 10 (excellent)	**Score**
PF ability to identify, assess, and summarize relevant data - consider baseline assessment, PDSA cycle measures and on-going monitoring	
PF ability to communicate effectively with QI team	
PF ability to encourage interactive problem solving and consensus building	
PF ability to encourage a diverse QI team including key personnel	
PF ability to encourage and sustain change efforts eg, shares info across practices, celebrates etc.	
PF ability to tailor facilitation activities to practice needs and circumstances	

6.3 MEASURES TO MINIMIZE BIAS: RANDOMIZATION AND BLINDING

Bias: Although most providers will be from St Louis metropolitan area, they will be drawn from the bi-state area. We will gather information to describe participants and eligible non-participants to assess selection bias and allow others to assess the relevance of our findings to their situation.

Randomization: We will stratify practices into 2-groups by the number of providers. (≤ 4 and ≥ 5) We will randomize within the strata using a block design.

Blinding: It is not possible to blind investigators or practices from group assignment. The RA and the data analyst will be blinded to group assignment and are responsible for assessment of the primary and secondary outcome measures of HPV vaccine use. Blinding is not necessary for baseline measurements as this will occur prior to randomization. Thus, baseline measures may be completed by other members of the study team. In addition, measurement bias will be minimized for the assessment of these outcomes by using a standardized approach for data collection and analysis, as well as quality checks.

6.4 STUDY INTERVENTION/EXPERIMENTAL MANIPULATION ADHERENCE

The facilitators will record the number and duration of visits to the practice, number of QI team meetings and attendees, and PDSA cycles undertaken.

To supplement these measures, guide the intervention and assess the intensity of the facilitation process, the facilitator will keep detailed field notes about practice observations and interactions that will be reviewed at weekly meetings.

7 STUDY INTERVENTION/EXPERIMENTAL MANIPULATION DISCONTINUATION AND PARTICIPANT DISCONTINUATION/WITHDRAWAL

7.1 DISCONTINUATION OF STUDY INTERVENTION/EXPERIMENTAL MANIPULATION

If new knowledge becomes available regarding the safety or efficacy of the HPV vaccine use, the study may be discontinued.

7.2 PARTICIPANT DISCONTINUATION/WITHDRAWAL FROM THE STUDY

At the discretion of the PI, study participants may be withdrawn for noncompliance. Providers are free to withdraw from participating in the study at any time upon request. Parent/caregiver participation is one-time only with no follow-up and is limited to completion of one questionnaire.

7.3 LOST TO FOLLOW-UP

N/A

8 STUDY ASSESSMENTS AND PROCEDURES

8.1 ENDPOINT AND OTHER NON-SAFETY ASSESSMENTS

Mixed methods of data collection will be used including audit of the electronic medical record (EMR) to extract routinely collected and previously recorded data, surveys, and field notes. The study team will follow the same measurement procedures for both study groups.

The primary and secondary outcomes (vaccine initiation and completion by age 13, respectively) will be assessed by audit of the EMR, completed by the research assistant (RA) and/or a member of the study team. To assess variation among providers in the same practice, we will sample records from each participating provider. Measurement will be completed over four 12-month time periods (baseline, 12, 24, and 36 months). For each measurement period, the physician's office will provide an electronic list of eligible patients with an office visit with each participating provider. For each practice, a member of the study team will randomly select a sample of 60 records per provider from this list for review by the RA and or other member of the study team. As much as possible the number of records will be balanced across providers. Data abstracted from the record will include date and type of index visit (well child, acute care, and other e.g., immunization), date of birth, date of vaccination for HPV vaccine, Tdap, and MCV, sex, race/ethnicity and insurance status. Records will be de-identified for patient, provider and practice identifiers, and given a unique subject ID number.

Providers: At baseline and at 12- and 24-months, in addition to the EMR audit described above, all participating providers will complete a brief survey to assess their knowledge, attitudes and behaviors regarding HPV vaccination. The baseline survey will also assess demographic and other practices and physician-level factors that may affect intervention success and are needed to describe the study sample. The practice facilitator will keep detailed field notes about practice observations, contextual factors and practice change components, and will monitor the implementation process.

At the conclusion of the study, providers will have the opportunity to complete a survey assessing the intervention's outcomes. This survey, distributed through REDCap software, will include questions related to acceptability, appropriateness, and sustainability of the intervention.

Practices: At baseline, the project coordinator and/or a member of the study team will meet with the practice manager to complete a brief practice survey to describe the practice and current HPV vaccination procedures. HPV vaccination procedures will be reassessed in a similar manner at 12- and 24- months.

Following the conclusion of the intervention, intervention practices will be invited to participate in an exit interview with the PI to assess the providers and their staff's experiences in participating in the HPV vaccine intervention, and to get input from participants about potential improvements in the intervention. These interviews will be audio recorded, consent for recording will be verbal.

8.3 Adverse Events and Serious Adverse Events

8.3.1 DEFINITION OF ADVERSE EVENTS

The care being provided to patients by participating study practices is indicated and therefore the only potential adverse event is breach of confidentiality. Regular monitoring will occur and any breach of confidentiality will be reported to the IRB as required per institutional guidelines if necessary.

8.4 UNANTICIPATED PROBLEMS

8.4.1 DEFINITION OF UNANTICIPATED PROBLEMS

This protocol uses the definition of Unanticipated Problems as defined by the Office for Human Research Protections (OHRP). OHRP considers unanticipated problems involving risks to participants or others to include, in general, any incident, experience, or outcome that meets all the following criteria:

- Unexpected in terms of nature, severity, or frequency given (a) the research procedures that are described in the study-related documents, such as the IRB-approved research protocol; and (b) the characteristics of the subject population being studied.
- Related or possibly related to participation in the research.
- Suggests that the research places participants or others at a greater risk of harm (including physical, psychological, economic, or social harm) than was previously known or recognized.

8.4.2 UNANTICIPATED PROBLEMS REPORTING

The PI will report unanticipated problems to WU IRB as required per institutional guidelines if necessary.

9 STATISTICAL CONSIDERATIONS

9.1 STATISTICAL HYPOTHESES

- Primary Efficacy Endpoint(s):

We hypothesize that the 2-year intervention will increase the percentage of preteens who receive HPV vaccine before their 13th birthday by at least 13 percentage points at 24 months compared to control, and benefits will be sustained at 36 months.

Alternatively, our null hypothesis is that there will be no difference in HPV vaccine use between the intervention and control groups.

9.2 SAMPLE SIZE DETERMINATIONS

AIM 1: We will use a cluster randomized design. Study outcomes will be measured at the level of the patient using a cross-sectional design. The sample size determination is based on the primary outcome - initiation of HPV vaccination by age 13. Effectiveness is derived from the difference in the proportion of eligible preteens in the intervention and control groups 24 months after the intervention, and long-term maintenance is derived from the difference in these measures 36 months after intervention. In the control group, national data suggest the baseline vaccine initiation proportion should be 0.4. We expect the vaccine initiation proportion in the control group in this age group will not change over time, and intervention group will have a similar baseline initiation rate to the control group, but will increase by at least 0.13 by 24 months. Thus, the hypothesis to test is H_0: p=0.4 vs. $H1$: p=0.53.

Our study has a three level hierarchical structure: children are clustered within providers which are clustered within practices. Thus, there exists correlations for children seen by the same provider, as well as providers working in the same practice. We use random effects to capture the intra-class correlation at the provider and the practice levels. From our preliminary data, the estimate for random effect variance at the practice level is very close to 0, and that at the provider level is 0.75^2 (corresponding to an ICC of 0.15). This suggests very little heterogeneity at the practice level, but significant heterogeneity at the provider level. Since there is no ready-to-use software for power calculation for clustered trial in the three level hierarchical structure, we conduct the power calculation by simulation.

In the simulation study, we set $\sigma_a^2 = .01$ for the random effect at the practice level and $\sigma_b^2 = .75^2$ for the random effect at the provider level. We choose the value of β so that the proportion of HPV vaccine rate is 0.4 for control and 0.53 for the intervention. We assume there are 4 physicians in each practice, each physician sees 30 children, resulting a total of 120 patients in each practice. The sample size to calculate is the number of practices. The simulation study is carried out in R, using function *glmer()* in the *mle4* package for estimation of the generalized linear mixed effects model (1) below.

Our original simulation results showed that 10 practices in the control arm and 10 practices in the intervention arm (a total of 120*20=2,400 children) would achieve a power over 0.80, to reject the null hypothesis under the significance level of 0.05. To address variation in the number of providers/practice, and to account for inclusion of more providers than anticipated (95 vs 80), we have chosen to increase

the sample size to 60 patients/provider, to ensure adequate power to detect a meaningful difference between the control group and the intervention group.

We also considered different baseline initiation rates in the control group in the range of 0.4 to 0.6. Our sample size of 10 practices in each arm has sufficient power to detect a difference in the rate of 0.12 when the baseline rate is 0.5 (i.e., $H_0: p = 0.5$ vs. $H_1: p = 0.62$), and 0.12 when the baseline rate is 0.6 (i.e., $H_0: p = 0.6$ vs. $H_1: p = 0.72$). In summary, the sample size of 20 practices provides adequate power to detect a 13% or larger effect.

To date, the literature suggests increased vaccination in 13-17 year-olds, but little year-to-year variation in vaccine outcomes in 11-12 year-olds, and so we have assumed no change in control group outcomes for our calculations. Finally, we considered the unlikely event of a 5% increase in vaccine initiation in the target group. In this case, our calculations suggest that a sample of 10/group would provide over 80% power to detect an 18% increase in the intervention group.

AIM 2: The joint significance test[79] will be used to test if the mediation effect is significant. The p-value from the joint significance test is the maximum of the p-values in testing $\alpha1$=0 and γ=0 separately, that is, it requires that both $\alpha1$ and γ are significant simultaneously. We thus use a Bonferroni's correction to set the significance level to be 0.05/2=0.025 for each test. We expect the power in testing γ=0 is lower since the outcome is a binary variable, while the mediator is a continuous or categorical variable which can yield more efficiency and higher power. Thus, we will repeat our simulation study in Aim 1 for power calculation, but use the alpha level of 0.025 instead of 0.05. Our sample size of 10 practices in each arm has sufficient power to detect a difference in the rate of 0.14 when the baseline rate is 0.4, and 0.13 when the baseline rate is 0.5 or 0.6. In summary, the sample size of 20 practices provides adequate power to detect a 14% or larger effect.

9.3 POPULATIONS FOR ANALYSES

Data analyses will adhere to the intention-to-treat principle, allowing 12-months grace period to implement practice changes. The primary analysis will compare the overall proportion of eligible preteens who initiated and completed HPV vaccination in the intervention and control practices at 24 months, using Year 2 measurements for vaccine use. Maintenance of change will be assessed using Year 3 measurements.

9.4 STATISTICAL ANALYSES

9.4.1 GENERAL APPROACH

All main data analyses will adhere to the intention-to-treat principle, and a probability of $P < 0.05$ will be used to establish statistical significance (2-tailed test). Before we perform complicated statistical modeling, we will conduct exploratory data analysis. For continuous variables, we will examine their distribution, and summarize them with mean/standard deviation if data are reasonably normally

distributed, or with median/interquartile range if data are skewed. We will use Rosner's outlier detection routine. Categorical data will be summarized by proportions. Simple comparison of continuous variables between intervention and control group will be performed using two sample t test or non-parametric Wilcoxon test. Simple comparison of categorical variable will be performed using chi-square test or fisher exact test where appropriate.

9.4.2 ANALYSIS OF THE PRIMARY ENDPOINT(S)

Data analyses will adhere to the intention-to-treat principle, allowing 12-months grace period to implement practice changes. The primary analysis will compare the overall proportion of eligible preteens who initiated and completed HPV vaccination in the intervention and control practices at 24 months, using Year 2 measurements for vaccine use. Maintenance of change will be assessed using Year 3 measurements. The analytic approach is to use a multi-level random effects logistic model to account for the hierarchical structure: children clustered within providers clustered within practices.

Define by y_{ijk} as the binary variable of HPV vaccine for the kth child of provider j of practice i. There are 3 hierarchical levels: children clustered within providers clustered within practices. We use random effects to capture the intra-class correlation at the provider and the practice levels. We assume a multi-level random effects logistic model (a generalized linear mixed model):

$$\text{logit } P(y_{ijk}=1)=\beta_0+trt_{ijk}\beta_1+a_i+b_{ij} \qquad (1)$$

where trt_{ijk} is the treatment indicator for practice i, and β_1 is the treatment effect. We use random effects a_i and b_{ij} to describe heterogeneity in the practice level and provider level, and assume a_i~$(0,\sigma_a^2)$ and $b_{ij} \sim N(0,\sigma_b^2)$ are independent. The practice level random intercept is used to accommodate extra-variation in outcome measurements due to clustering of providers in a practice, and the provider level random intercept is used to accommodate extra-variation in outcome measurements due to clustering of individual patients within a provider.
As secondary analysis, we can add other covariates in the model, e.g., gender, category of practice (urban vs. rural), vaccine rate at baseline for a provider or practice, etc. Interaction of treatment and these covariates can be included in the model to study the moderating effect of these risk factors. The interaction of random effects (both at the practice and provider levels) and covariates can be added in the model as well.

Missing Data: As in any other study, we expect some missing data in our study. If missing data affects the validity of statistical inference, we will follow Little and Rubin to deal with this problem.[78] If the assumption of "missing at random" holds, this approach will avoid bias and preserves the study power. Specifically, multiple imputed completed datasets, say 5, will be generated through parametric (or nonparametric) Bayesian models. For each imputed dataset, the standard statistical method is applied to obtain the parameter estimates with the estimated variance and covariance. Then, these parameter estimates with their variance and covariance estimates can be used to generate overall parameter estimates and their standard errors.

9.4.3 ANALYSIS OF THE SECONDARY ENDPOINT(S)

We will use mediation analysis to test whether the provider's capability and provider's beliefs about their capabilities mediate the intervention's effect on vaccination outcomes.

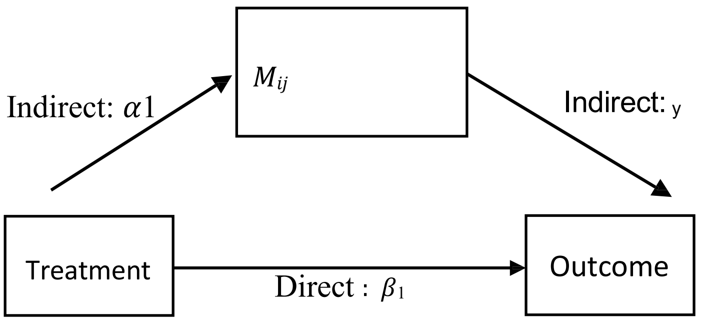

Define the provider's beliefs about their capabilities by M_{ij}, we can write the model as

$$M_{ij}=\alpha_0+trt_{ijk}\alpha_1+c_i+e_{ij} \quad (2)$$

$$\text{logit } P(y_{ijk}=1)=\beta_0+trt_{ijk}\beta_1+M_{ij}\gamma+a_i+b_{ij} \quad (3)$$

We are interested in testing the hypothesis $H_o{:}\alpha_1\gamma = 0$ vs. $H_1{:}\alpha_1\gamma \neq 0$. The joint significance test[79] can be used to test the significance of the mediation effect. We include random effect c_i to capture the correlation of the provider's capabilities within the same practice. The indirect effect, i.e., the effect of intervention to the outcome through the mediator is specified as the product of α_1 and γ. The direct effect of treatment on outcome is denoted by β_1. We can use a similar model for provider's capability.

Finally, we can also include the interaction of treatment and mediator in Model (3), to test whether the mediator is also a moderator of treatment in the "mediator moderator" model.

We will use mediation analysis to test whether the intervention effect on vaccination outcomes is mediated by the degree of practice change as measured by an 18-item HPV vaccine delivery score.

For implementation outcomes (fidelity, acceptability, appropriateness), we will complete a descriptive analysis. Parental satisfaction will be modeled in a three-level (practice, provider, and child's parent) linear mixed effects model. Descriptive statistics will be given on implementation fidelity, the acceptability and appropriateness of the intervention for providers, parental satisfaction with care, and cost. Of note, fidelity score is measured at the practice level every 6 months in the intervention group only. Parental satisfaction is measured in both study groups at baseline and 24 months. Provider acceptance and appropriateness is measured in intervention group at 24 months.

Denote by y_{it} the fidelity score of practice i at time t. Repeated measures of fidelity score will be modeled by mixed effects models.

$$y_{it} = \beta_0 + \beta_1 t + a_i + e_{ij}, \quad (4)$$

Where β_1 is the slope for the temporal change in the fidelity score, a_i is the random intercept for practice i, and e_{ij} is the error term. By testing $\beta_1 = 0$, we can see if there is a significant increase in the fidelity score over time in the intervention group. We can add a random slope of time in this model to capture the heterogeneity in such a temporal change.

As another example, parental satisfaction will be modeled in a three-level (practice, provider, and child's parent) linear mixed effects model:

$$y_{ijk} = \beta_0 + trt_{ijk}\beta_1 + a_i + b_{ij} + e_{ijk}$$

For cost, it is beyond the scope of this project to complete a cost-effectiveness analysis that includes all long-term related social costs and benefits (e.g., prevention of cancer, reducing costs of secondary prevention activities). Rather, we will prospectively assess the cost to replicate the intervention in practice.[77] To generalize beyond Missouri, geographic wage data from the Bureau of Labor Statistics, and average Medicaid allowable rates will establish a minimum on cost reimbursement

9.4.4 SAFETY ANALYSES

N/A

9.4.5 BASELINE DESCRIPTIVE STATISTICS

Before we perform complicated statistical modeling, we will conduct exploratory data analysis. For continuous variables, we will examine their distribution, and summarize them with mean/standard deviation if data are reasonably normally distributed, or with median/interquartile range if data are skewed. We will use Rosner's outlier detection routine. Categorical data will be summarized by proportions. Simple comparison of continuous variables between intervention and control group will be performed using two sample t test or non-parametric Wilcoxon test. Simple comparison of categorical variable will be performed using chi-square test or fisher exact test where appropriate.

9.4.6 PLANNED INTERIM ANALYSES

N/A

9.4.7 SUB-GROUP ANALYSES

We plan two subgroup analyses to explore intervention effectiveness for girls and boys, and for those with Medicaid insurance compared to other health insurance.
As secondary analysis, we can add other covariates in the model used in the primary analysis, e.g., gender, category of practice (urban vs. rural), vaccine rate at baseline for a provider or practice, etc.

Interaction of treatment and these covariates can be included in the model to study the moderating effect of these risk factors. The interaction of random effects (both at the practice and provider levels) and covariates can be added in the model as well.

9.4.8 TABULATION OF INDIVIDUAL PARTICIPANT DATA

N/A

9.4.9 EXPLORATORY ANALYSES

As secondary exploratory analyses, we can add other covariates in the model used in the primary analysis, e.g., gender, category of practice (urban vs. rural), vaccine rate at baseline for a provider or practice, etc. Interaction of treatment and these covariates can be included in the model to study the moderating effect of these risk factors. The interaction of random effects (both at the practice and provider levels) and covariates can be added in the model as well.

10 SUPPORTING DOCUMENTATION AND OPERATIONAL CONSIDERATIONS

10.1 REGULATORY, ETHICAL, AND STUDY OVERSIGHT CONSIDERATIONS

10.1.2 STUDY DISCONTINUATION AND CLOSURE

This study may be temporarily suspended or prematurely terminated if there is sufficient reasonable cause. Written notification, documenting the reason for this occurrence will be provided by the suspending or terminating party to study practices, the investigator, funding agency and regulatory authorities. If the study is prematurely terminated or suspended, the PI will promptly inform participating practices, the IRB and NIH, and will provide reasons for the termination or suspension.

Circumstances that may warrant termination or stopping include but are not limited to:

- Determination of unexpected, significant, or unacceptable risk to participants
- Insufficient compliance of study staff to the protocol
- Data that are not sufficiently complete or evaluable
- Determination that the primary end point has been met
- Determination of futility

The study may resume once concerns about safety, protocol compliance and data quality area addressed, and satisfy the funding agency, sponsor, IRB, FDA, or other regulatory or oversight bodies such as the IRB.

10.1.6 SAFETY OVERSIGHT

As a safety monitoring committee is not required by the NIH for this low risk study, safety oversight will be under the direction of the Principal Investigator. Dr. Garbutt and the Co-Investigators will provide study oversight and monitor the conduct of the trial to ensure the safety of the participants and the validity and integrity of the data. Responsibilities include: 1) Review trial performance information such as accrual information, protocol violations, and unanticipated problems and 2) Review major proposed modifications to the study prior to their implementation (e.g., changing target sample size). They will meet at least quarterly to assess study progress and safety, and provide recommendations related to resolving any issues with the trial.

10.3 ABBREVIATIONS AND SPECIAL TERMS

Adverse Event	AE
Agency for Healthcare Research and Quality	AHRQ
American Board of Pediatrics	ABP
Centers for Disease Control and Prevention	CDC
Certificate of Confidentiality	COC
Clinical and Translational Science Award	CTSA
Code of Federal Regulations	CFR
Consolidated Framework for Implementation Research	CFIR
Consolidated Standards of Reporting Trials	CONSORT
Data Coordinating Center	DCC
Data Safety Monitoring Board	DSMB
Designing for Dissemination	D4D
Electronic Case Report Forms	eCRF
Electronic Medical Record	EMR
Ethics Committee	EC
Food and Drug Administration	FDA
Good Clinical Practice	GCP
Health Insurance Portability and Accountability Act	HIPAA
Healthcare Effectiveness Data and Information Set	HEDIS
Human Papilloma Virus	HPV
Independent Safety Monitor	ISM
Institutional Review Board	IRB
International Council on Harmonisation	ICH
Maintenance of Certification	MOC
Manual of Procedures	MOP
Meningococcal Vaccine	MCV
National Clinical Trial	NCT
National Health and Nutrition Examination Survey	NHANES
National Institutes of Health	NIH
Office for Human Research Protections	OHRP
Plan-Do-Study-Act	PDSA
Practice Based Research Network	PBRN
Principal Investigator	PI
Quality Assurance	QA
Quality Control	QC
Quality Improvement	QI
Randomized Control Trial	RCT
Research Assistant	RA

Research Electronic Data Capture	REDCap
Safety Monitoring Committee	SMC
Schedule of Activities	SOA
Serious Adverse Event	SAE
Standard Operating Procedure	SOP
Statistical Analysis Plan	SAP
Statistical Analysis software	SAS
Tetanus-Diphtheria-Pertussis	Tdap
Theoretical Domains Framework	TDF
Unanticipated Problem	UP
United States	US
Washington University	WU
Washington University School Of Medicine	WUSM
Washington University Pediatric and Adolescent Ambulatory Research Consortium	WUPAARC

10.4 PROTOCOL AMENDMENT HISTORY

Version	Date	Description of Change	Brief Rationale
1.02	12/20/2019	Section 6.3: Blinding not needed for assessment of baseline measures<br>Section 10.1.6: PI will be responsible for safety oversight rather than a safety monitoring committee	Baseline measures are completed prior to randomization.<br>The study is assessed as low risk and a safety monitoring committee is not required by the NIH.
1.03	1/10/2020	Sections 5.5 and 8.1: Removed #30 from the eligible sample list for parent surveys.	To obtain 30 parents/practice, we will need to invite a larger list of eligible parents.
1.04	2/10/2020	Sections 1.1 and 6.11: Added, all providers will receive baseline HPV vaccination rates.<br>Section 6.3: Added stratification to the block design for randomization.	In order to allow all providers to know the results of chart audits.<br>To balance the randomization process for number of providers in the practice.
1.05	3/24/2020	Section 5.5 and 8.1: Clarified who will send an email or text invitation for study participation to parents.	The email or text invitation will come from the study team, providers have the option to send an email or text to the parents.
1.05	3/24/2020	Section 1.2: Study Schema to reflect measurement for 160 preteens per practice	To maintain power and address the variability in the recruited practices (2-8 providers), we increased the sample size of patients.
1.05	3/24/2020	Section: 8.1 and 9.2: Increased sample size to 160 patients/practice.	To maintain power and address the variability in the recruited practices (2-8 providers).
1.06	5/13/2020	Eliminated the parent survey at baseline.	Due to the 2020 outbreak of Covid-19, it is not possible to obtain the necessary information for a parent survey at this time. The survey asks parents to recall their well-child visit for 2019, which would not be useful data at this time, and could add stress on to the providers.
1.06	5/13/2020	Added research study activities may be delivered by Zoom video conferencing.	Due to the 2020 outbreak of Covid-19, we included Zoom video conferencing as a way to

			implement study activities.
1.07	1/25/22	Increased sample size to 60patients/providers	Due to the participation of more providers than anticipated (95 vs 80) and because of variation among providers, our statistician has suggested to increase the sample size to 60 patients per provider to allow the power to detect a meaningful difference between the control group and intervention group.
1.08	6/22/2022	Eliminated the parent survey from the study.	Due to restrictions with obtaining parent email addresses from the EMR and individual practices it is not feasible to complete the parent survey.
1.3, 8.1	8/26/2022	Added the exit interview for the intervention practices.	To assess the providers and their staff's experiences in participating in the HPV vaccine intervention, and to get input from participants about potential improvements in the intervention.
1.3, 6.2.1	2/26/2023	Updated the fidelity score	**To assess the providers and their staff's experiences in participating in the HPV vaccine intervention, and to get input from participants about potential improvements in the intervention..** The fidelity score template was updated to prevent any subjective bias. The scoring timeline was updated from every 6 months to annually.
8.2024		Updated the Principal Investigator	Due to staffing changes, the study PI has been changed.

11 REFERENCES

1. Petrosky EY, Liu G, Hariri S, Markowitz LE. Human Papillomavirus Vaccination and Age at First Sexual Activity, National Health and Nutrition Examination Survey. *Clin Pediatr (Phila).* 2017;56(4):363-370.
2. Walker TY, Elam-Evans LD, Yankey D, et al. National, Regional, State, and Selected Local Area Vaccination Coverage Among Adolescents Aged 13–17 Years—United States, 2017. *Morbidity and Mortality Weekly Report.* 2018;67(33):909.
3. Healthy People 2020. http://healthypeople.gov. Accessed April 20, 2015.
4. Chesson HW, Ekwueme DU, Saraiya M, Dunne EF, Markowitz LE. The estimated impact of human papillomavirus vaccine coverage on the lifetime cervical cancer burden among girls currently aged 12 years and younger in the United States. *Sexually transmitted diseases.* 2014;41(11):656-659.
5. Markowitz LE, Dunne EF, Saraiya M, et al. Human papillomavirus vaccination: recommendations of the Advisory Committee on Immunization Practices (ACIP). *Morbidity and Mortality Weekly Report: Recommendations and Reports.* 2014;63(5):1-30.
6. Walker TY, Elam-Evans LD, Singleton JA, et al. National, Regional, State, and Selected Local Area Vaccination Coverage Among Adolescents Aged 13-17 Years - United States, 2016. *MMWR Morb Mortal Wkly Rep.* 2017;66(33):874-882.
7. Stokley S, Jeyarajah J, Yankey D, et al. Human papillomavirus vaccination coverage among adolescents, 2007–2013, and postlicensure vaccine safety monitoring, 2006–2014—United States. *Morbidity and Mortality Weekly Report.* 2014;63(29):620-624.
8. Chesson HW, Ekwueme DU, Saraiya M, Watson M, Lowy DR, Markowitz LE. Estimates of the annual direct medical costs of the prevention and treatment of disease associated with human papillomavirus in the United States. *Vaccine.* 2012;30(42):6016-6019.
9. Fu LY, Bonhomme L-A, Cooper SC, Joseph JG, Zimet GD. Educational interventions to increase HPV vaccination acceptance: a systematic review. *Vaccine.* 2014;32(17):1901-1920.
10. Edwards KM, Hackell JM, Diseases CoI, Practice Co, Medicine A. Countering vaccine hesitancy. *Pediatrics.* 2016:e20162146.
11. Gilkey MB, Calo WA, Marciniak MW, Brewer NT. Parents who refuse or delay HPV vaccine: Differences in vaccination behavior, beliefs, and clinical communication preferences. *Human vaccines & immunotherapeutics.* 2017;13(3):680-686.
12. Smulian EA, Mitchell KR, Stokley S. Interventions to increase HPV vaccination coverage: A systematic review. *Hum Vaccin Immunother.* 2016;12(6):1566-1588.
13. Niccolai LM, Hansen CE. Practice- and Community-Based Interventions to Increase Human Papillomavirus Vaccine Coverage: A Systematic Review. *JAMA pediatrics.* 2015;169(7):686-692.
14. Walling EB, Benzoni N, Dornfeld J, et al. Interventions to improve HPV vaccine uptake: a systematic review. *Pediatrics.* 2016;138(1):e20153863.
15. Brewer NT, Hall ME, Malo TL, Gilkey MB, Quinn B, Lathren C. Announcements Versus Conversations to Improve HPV Vaccination Coverage: A Randomized Trial. *Pediatrics.* 2017;139(1).
16. Paskett ED, Krok-Schoen JL, Pennell ML, et al. Results of a Multilevel Intervention Trial to Increase Human Papillomavirus (HPV) Vaccine Uptake among Adolescent Girls. *Cancer Epidemiol Biomarkers Prev.* 2016;25(4):593-602.
17. Zimmerman RK, Moehling KK, Lin CJ, et al. Improving adolescent HPV vaccination in a

randomized controlled cluster trial using the 4 Pillars practice Transformation Program. *Vaccine.* 2017;35(1):109-117.

18. Fiks AG, Grundmeier RW, Mayne S, et al. Effectiveness of decision support for families, clinicians, or both on HPV vaccine receipt. *Pediatrics.* 2013;131(6):1114-1124.
19. Perkins RB, Zisblatt L, Legler A, Trucks E, Hanchate A, Gorin SS. Effectiveness of a provider-focused intervention to improve HPV vaccination rates in boys and girls. *Vaccine.* 2015;33(9):1223-1229.
20. Gilkey MB, McRee AL. Provider communication about HPV vaccination: A systematic review. *Hum Vaccin Immunother.* 2016;12(6):1454-1468.
21. Dempsey AF, Pyrznawoski J, Lockhart S, et al. Effect of a health care professional communication training intervention on adolescent human papillomavirus vaccination: A cluster randomized clinical trial. *JAMA pediatrics.* 2018:e180016.
22. Holman DM, Benard V, Roland KB, Watson M, Liddon N, Stokley S. Barriers to human papillomavirus vaccination among US adolescents: a systematic review of the literature. *JAMA pediatrics.* 2014;168(1):76-82.
23. Perkins RB, Clark JA. What affects human papillomavirus vaccination rates? A qualitative analysis of providers' perceptions. *Women's health issues : official publication of the Jacobs Institute of Women's Health.* 2012;22(4):e379-386.
24. National Ambulatory Medical Care Survey – Community Health Centers: 2012 State and National Summary Tables. 2012; https://www.cdc.gov/nchs/data/ahcd/namcs_summary/2012_namcs_chc_web_tables.pdf Accessed 3/9/18.
25. Freed GL, Moran LM, Van KD, Leslie LK, Pediatrics RACotABo. Current workforce of general pediatricians in the United States. *Pediatrics.* 2016;137(4):e20154242.
26. Hough DE, Liu K, Gans DN. Size matters: the impact of physician practice size on productivity. *Unpublished manuscript Medical Group Management Association (CO).* 2011.
27. Solberg LI. Improving medical practice: a conceptual framework. *The Annals of Family Medicine.* 2007;5(3):251-256.
28. Crabtree BF, Nutting PA, Miller WL, et al. Primary care practice transformation is hard work: insights from a 15-year developmental program of research. *Medical care.* 2011;49(Suppl):S28.
29. Stange KC, Goodwin MA, Zyzanski SJ, Dietrich AJ. Sustainability of a practice-individualized preventive service delivery intervention. *American journal of preventive medicine.* 2003;25(4):296-300.
30. Martin J, Avant R, Bowman M, et al. The Future of Family Medicine: a collaborative project of the family medicine community. *Annals of family medicine.* 2004;2(2 Suppl 1):S3-32.
31. Nutting PA, Crabtree BF, Stewart EE, et al. Effect of facilitation on practice outcomes in the National Demonstration Project model of the patient-centered medical home. *The Annals of Family Medicine.* 2010;8(Suppl 1):S33-S44.
32. Dickinson WP, Dickinson LM, Nutting PA, et al. Practice facilitation to improve diabetes care in primary care: a report from the EPIC randomized clinical trial. *The Annals of Family Medicine.* 2014;12(1):8-16.
33. Miller WL, Crabtree BF, Nutting PA, Stange KC, Jaén CR. Primary care practice development: a relationship-centered approach. *The Annals of Family Medicine.* 2010;8(Suppl 1):S68-S79.
34. McNellis RJ, Genevro JL, Meyers DS. Lessons learned from the study of primary care transformation. In: Annals Family Med; 2013.
35. Goodson P, Smith MM, Evans A, Meyer B, Gottlieb NH. Maintaining prevention in practice:

survival of PPIP in primary care settings. *American Journal of Preventive Medicine.* 2001;20(3):184-189.
36. Wei EK, Ryan CT, Dietrich AJ, Colditz GA. Improving colorectal cancer screening by targeting office systems in primary care practices: disseminating research results into clinical practice. *Archives of internal medicine.* 2005;165(6):661-666.
37. Dietrich AJ, O'Connor GT, Keller A, Carney PA, Levy D, Whaley FS. Cancer: improving early detection and prevention. A community practice randomised trial. *Bmj.* 1992;304(6828):687-691.
38. Dietrich AJ, Olson AL, Hill Sox C, Woodruff Winchell C, Grant-Petersson J, Collison DW. Sun protection counseling for children: primary care practice patterns and effect of an intervention on clinicians. *Archives of family medicine.* 2000;9(2):155.
39. Goodson P, Gottlieb NH, Smith MM. Put prevention into practice. *American journal of preventive medicine.* 1999;17(1):73-78.
40. Goodwin MA, Zyzanski SJ, Zronek S, et al. A clinical trial of tailored office systems for preventive service delivery. *American journal of preventive medicine.* 2001;21(1):20-28.
41. Bordley WC, Margolis PA, Stuart J, Lannon C, Keyes L. Improving preventive service delivery through office systems. *Pediatrics.* 2001;108(3):e41-e41.
42. Damschroder LJ, Aron DC, Keith RE, Kirsh SR, Alexander JA, Lowery JC. Fostering implementation of health services research findings into practice: a consolidated framework for advancing implementation science. *Implement Sci.* 2009;4:50.
43. Cane J, O'Connor D, Michie S. Validation of the theoretical domains framework for use in behaviour change and implementation research. *Implement Sci.* 2012;7:37.
44. Atkins L, Francis J, Islam R, et al. A guide to using the Theoretical Domains Framework of behaviour change to investigate implementation problems. *Implement Sci.* 2017;12(1):77.
45. Michie S, Johnston M, Abraham C, et al. Making psychological theory useful for implementing evidence based practice: a consensus approach. *Qual Saf Health Care.* 2005;14(1):26-33.
46. French SD, Green SE, O'Connor DA, et al. Developing theory-informed behaviour change interventions to implement evidence into practice: a systematic approach using the Theoretical Domains Framework. *Implement Sci.* 2012;7:38.
47. Garbutt JM, Dodd S, Walling E, Lee AA, Kulka K, Lobb R. Theory-based development of an implementation intervention to increase HPV vaccination in pediatric primary care practices. *Implementation Science.* 2018;13(1):45.
48. Garbutt JM, Dodd S, Walling E, Lee AA, Kulka K, Lobb R. Barriers and facilitators to HPV vaccination in primary care practices: a mixed methods study using the Consolidated Framework for Implementation Research. *BMC family practice.* 2018;19(1):53.
49. Davis DA, Taylor-Vaisey A. Translating guidelines into practice: a systematic review of theoretic concepts, practical experience and research evidence in the adoption of clinical practice guidelines. *Canadian Medical Association Journal.* 1997;157(4):408-416.
50. Greco PJ, Eisenberg JM. Changing physicians' practices. In: Mass Medical Soc; 1993.
51. Aarons GA, Sklar M, Mustanski B, Benbow N, Brown CH. "Scaling-out" evidence-based interventions to new populations or new health care delivery systems. *Implementation Science.* 2017;12(1):111.
52. Garbutt JM, Yan Y, Highstein G, Strunk RC. A cluster-randomized trial shows telephone peer coaching for parents reduces children's asthma morbidity. *Journal of Allergy and Clinical Immunology.* 2015;135(5):1163-1170. e1162.
53. Tabak RG, Chambers DA, Hook M, Brownson RC. Dissemination and Implementation

Research in Health: Translating Science to Practice In: Oxford University Press; 2018.
54. Michie S, van Stralen MM, West R. The behaviour change wheel: a new method for characterising and designing behaviour change interventions. *Implement Sci.* 2011;6:42.
55. Finer LB, Philbin JM. Sexual initiation, contraceptive use, and pregnancy among young adolescents. *Pediatrics.* 2013;131(5):886-891.
56. Copen CE, Chandra A, Martinez G. Prevalence and timing of oral sex with opposite-sex partners among females and males aged 15-24 years: United States, 2007-2010. *Natl Health Stat Report.* 2012(56):1-14.
57. Cavazos-Rehg PA, Krauss MJ, Spitznagel EL, et al. Age of sexual debut among US adolescents. *Contraception.* 2009;80(2):158-162.
58. Jena AB, Goldman DP, Seabury SA. Incidence of sexually transmitted infections after human papillomavirus vaccination among adolescent females. *JAMA internal medicine.* 2015;175(4):617-623.
59. Bednarczyk RA, Davis R, Ault K, Orenstein W, Omer SB. Sexual activity-related outcomes after human papillomavirus vaccination of 11- to 12-year-olds. *Pediatrics.* 2012;130(5):798-805.
60. Romanowski B, Schwarz TF, Ferguson L, et al. Sustained immunogenicity of the HPV-16/18 AS04- adjuvanted vaccine administered as a two-dose schedule in adolescent girls: five-year clinical data and modeling predictions from a randomized study. *Human vaccines & immunotherapeutics.* 2016;12(1):20- 29.
61. CDC. Human Papillomavirus (HPV) Resources for Health Professionals. https://www.cdc.gov/hpv/hcp/resources.html. Accessed March 9th, 2018.
62. Scherr C, Augusto B, Ali K, Malo T, Vadaparampil S. Provider-reported acceptance and use of the Centers for Disease Control and Prevention messages and materials to support HPV vaccine recommendation for adolescent males. *Vaccine.* 2016;34(35):4229-4234.
63. Margolis PA, Lannon CM, Stuart JM, Fried BJ, Keyes-Elstein L, Moore DE, Jr. Practice based education to improve delivery systems for prevention in primary care: randomised trial. *BMJ.* 2004;328(7436):388.
64. Pink DH. *Drive: The Suprising Truth About What Motivates Us.* 375 Hudson Street, New York, New York 10024, USA: Riverhead Books, Penguin Group (USA) Inc.; 2009.
65. Ruhe MC, Carter C, Litaker D, Stange KC. A systematic approach to practice assessment and quality improvement intervention tailoring. *Quality Management in Healthcare.* 2009;18(4):268-277.
66. Meropol SB, Schiltz NK, Sattar A, et al. Practice-tailored facilitation to improve pediatric preventive care delivery: a randomized trial. *Pediatrics.* 2014;133(6):e1664-e1675.
67. Huijg JM, Gebhardt WA, Dusseldorp E, et al. Measuring determinants of implementation behavior: psychometric properties of a questionnaire based on the theoretical domains framework. *Implementation Science.* 2014;9(1):33.
68. Malo TL, Hall ME, Brewer NT, Lathren CR, Gilkey MB. Why is announcement training more effective than conversation training for introducing HPV vaccination? A theory-based investigation. *Implementation Science.* 2018;13(1):57.
69. Coalition IA. Suggestions to improve your immunization services. 2006; http://www.immunize.org/catg.d/p2045.pdf.
70. Cohen DJ, Crabtree BF, Etz RS, et al. Fidelity versus flexibility: translating evidence-based research into practice. *American journal of preventive medicine.* 2008;35(5):S381-S389.
71. Shelley DR, Ogedegbe G, Anane S, et al. Testing the use of practice facilitation in a cluster randomized stepped-wedge design trial to improve adherence to cardiovascular disease

prevention guidelines: HealthyHearts NYC. *Implementation Science.* 2016;11(1):88.
72. Baskerville NB, Liddy C, Hogg W. Systematic review and meta-analysis of practice facilitation within primary care settings. *The Annals of Family Medicine.* 2012;10(1):63-74.
73. Abry T, Hulleman CS, Rimm-Kaufman SE. Using indices of fidelity to intervention core components to identify program active ingredients. *American Journal of Evaluation.* 2015;36(3):320-338.
74. Chamberlain P, Brown CH, Saldana L. Observational measure of implementation progress in community based settings: the stages of implementation completion (SIC). *Implementation Science.* 2011;6(1):116.
75. Weiner BJ, Lewis CC, Stanick C, et al. Psychometric assessment of three newly developed implementation outcome measures. *Implementation Science.* 2017;12(1):108.
76. Moss JL, Reiter PL, Rimer BK, Brewer NT. Collaborative patient-provider communication and uptake of adolescent vaccines. *Social Science & Medicine.* 2016;159:100-107.
77. Ritzwoller DP, Sukhanova A, Gaglio B, Glasgow RE. Costing Behavioral Interventions: A Practical Guide to Enhance Translation. *Annals of Behavioral Medicine.* 2009;37(2):218-227.
78. Little RJ, Rubin DB. *Statistical analysis with missing data.* Vol 333: John Wiley & Sons; 2002
79. Taylor, A., MacKinnon, D. and Tein, J. (2008). Tests of the three-path mediated effect. *Organizational Research Methods*, 11, 241-269.
