# Supplementary material for: A Multicomponent Strategy to Increase Human Papillomavirus Vaccination Rates in Primary Care: A Cluster Randomized Clinical Trial
Source: JAMA Netw Open. 2026 Feb 26;9(2):e260049. doi: 10.1001/jamanetworkopen.2026.0049 (PMC12947024; doi:10.1001/jamanetworkopen.2026.0049)
Supplement: Supplement 2. — eTable 1. Examples of Practice Facilitation Support eTable 2. Suggested Responses to Parent/Caregiver Questions eFigure 1. Key Elements of Communication Strategy eTable 3. Number of Clinicians and Patients by Practice at 12, 24, and 36 Months eTable 4. Intervention Effects on HPV Vaccination Initiation and Completion by Study Year: Percentage Point Differences eFigure 2. HPV Vaccination Initiation and Completion by Patient and Clinician Characteristics eTable 5. Clinician Characteristics Between Responders and Nonresponders Within Each Group eReferences. [file jamanetwopen-e260049-s002.pdf]

Supplementary Online Content

Wang R, Liu L, Dodd S, et al. A multicomponent strategy to increase human papillomavirus vaccination rates in primary care: a cluster randomized clinical trial. *JAMA Netw Open.* 2026;9(2):e260049. doi:10.1001/jamanetworkopen.2026.0049

eTable 1. Examples of Practice Facilitation Support
eTable 2. Suggested Responses to Parent/Caregiver Questions
eFigure 1. Key Elements of Communication Strategy
eTable 3. Number of Clinicians and Patients by Practice at 12, 24, and 36 Months
eTable 4. Intervention Effects on HPV Vaccination Initiation and Completion by Study Year: Percentage Point Differences
eFigure 2. HPV Vaccination Initiation and Completion by Patient and Clinician Characteristics
eTable 5. Clinician Characteristics Between Responders and Nonresponders Within Each Group
eReferences.

This supplementary material has been provided by the authors to give readers additional information about their work.

© 2026 Wang R et al. *JAMA Network Open.*

eTable 1. Examples of Practice Facilitation Support

Change Ideas	Desired Outcomes	Identified Barriers	Facilitation Strategies	Tailoring Examples
Target lower age of initiation	• Increased initiation rates;<br>• Increased completion rates;<br>• Standardized procedures for vaccine initiation	• Staff turnover;<br>• High levels of stress due to COVID;<br>• Operational COVID restrictions;<br>• Difficulty retrieving reports from electronic health record (EHR)	• Clinician interview assessments;<br>• Showed a flow chart that compared practice's rates amongst other participating practices;<br>• Audit and feedback to motivate change;<br>• Provided new research recommendations for HPV;<br>• Guided practices to find solutions to overcome obstacles while trying to accomplish their goals;<br>• Worked with EHR information technology (IT) practice managers or clinicians to create a practice data report	• Bundled HPV with other vaccines;<br>• Utilized HPV awareness posters and brochures in practice;<br>• Use of communication strategy with younger age;<br>• Key quality improvement (QI) team members providing solutions for change ideas
Consistent messaging across practice for HPV initiation	• Increased initiation rates;<br>• Skilled and confident workforce;<br>• Communication among clinicians within practice	• Staff turnover;<br>• Decrease in office visits due to COVID rates	• Provided training for clinicians and staff;<br>• Role play;<br>• Provided educational resources	• Strong recommendation for HPV;<br>• Clinicians give educational resource to any hesitant parent (brochure);<br>• Practice facilitator led group discussion for staff and clinicians to understand HPV messaging and process
Administer 2nd dose at other visit types	• Increased completion rates;<br>• Maximized vaccine opportunities;<br>• Standardized procedures for vaccine completion;<br>• Skilled and confident workforce	• COVID vaccine rollout;<br>• Parent vaccine hesitancy;<br>• Decrease in well-child visits due to COVID;<br>• Staff turnover;<br>• New EHR system	• Utilized Maintenance of Certification Part 4 credit as motivation for new change ideas;<br>• Encouraged/motivated practices to move forward to new change ideas when they had maintained previous change ideas or goals;<br>• Provided project management and organizational support;<br>• Supported practices with information and changes outside of project parameters	• Audit and feedback to motivate change;<br>• Cross-pollination among clinicians within the practice/project;<br>• Re-train/training because of high staff turnover;<br>• Schedule vaccine-only visits;<br>• Scheduling next visit in patient rooms;<br>• Check-out sheets;<br>• Implemented vaccine reminders

© 2026 Wang R et al. *JAMA Network Open.*

Change Ideas	Desired Outcomes	Identified Barriers	Facilitation Strategies	Tailoring Examples
Process/System for scheduling appointments	• Maximizing vaccine opportunities;<br>• Targeting gap between initiation and completion;<br>• Increased number of well-child visits;<br>• Increased number of vaccine-only visits	• High volume of sick patients;<br>• New EHR system;<br>• COVID vaccine rollout;<br>• Staff turnover;<br>• Lack of training time;<br>• High levels of stress due to COVID;<br>• Lack of patients in office;<br>• Lack of clinician adaptability;<br>• Operational COVID restrictions	• Guided practices to find solutions to overcome obstacles;<br>• Listened to clinician/staff COVID concerns;<br>• Reviewed monthly data to identify gaps in process;<br>• Cross-pollination to provide options;<br>• Met practices where they were and were adaptable and flexible;<br>• Provided project management and organizational support;<br>• Worked with EHR IT, practice managers, or clinicians to create a practice data report	• Implementing vaccine reminder system in EHR for staff/clinicians;<br>• Clinicians/staff review chart before every visit with a patient for vaccinations;<br>• Audit and feedback to motivate change;<br>• Implemented vaccine reminders;<br>• Key QI team members providing solutions for change ideas;<br>• Check-out sheets

© 2026 Wang R et al. *JAMA Network Open.*

eTable 2. Suggested Responses to Parent/Caregiver Questions

*Ask for permission to continue conversation on HPV vaccination prior to engagement with dialogue below: "I hear your concerns. Let me see if I can help you…" If parent/caregiver agrees, "It sounds like you're questioning that…" *

Question	Suggested Response[1,2]
HPV vaccine not required for school	"Yes, that is true it is not a required vaccine; however, it is our best tool for cancer prevention."
Too young	"Completing the series before the age of 13 helps to ensure your child is protected from HPV-related cancers before they are ever exposed to the virus."
My child won't get HPV	"HPV is a very common virus – 8 out of 10 people will get HPV in their lifetime."
Safety and efficacy	"The HPV vaccine has been tested and found to be safe, long-lasting, and effective at preventing cancer."<br>*OR*<br>"Hundreds of millions of doses have been given worldwide with no serious safety concerns identified."
Duration of protection	"HPV vaccine is long-lasting. Studies have followed vaccinated individuals for over 15 years and have found no evidence of decreasing vaccine effectiveness."
Earlier sex	"Research shows that getting the HPV vaccine and starting to have sex are not related."<br>*OR*<br>"New research shows that HPV can be contracted from non-sexual exposure too."
Boys getting HPV	"HPV is a very common virus – 8 out of 10 people will get HPV in their lifetime."<br>*OR*<br>"HPV infection can cause cancer in males, too."

© 2026 Wang R et al. *JAMA Network Open.*

eFigure 1. Key Elements of Communication Strategy

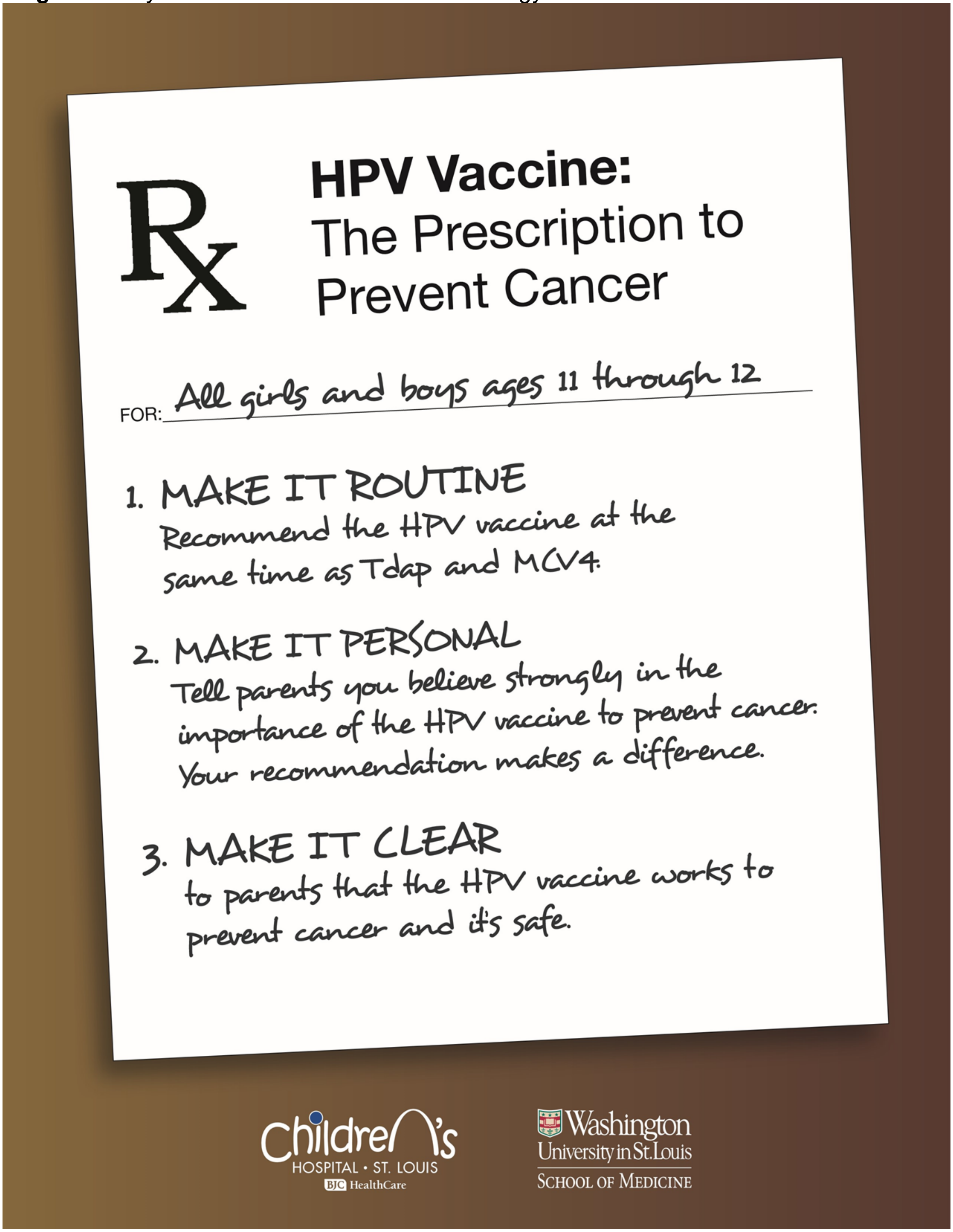

© 2026 Wang R et al. *JAMA Network Open*.

eFigure 1 (continued)

Rx HPV Vaccine: The Prescription to Prevent Cancer

1. Strongly recommend HPV vaccine **the same way you do all other vaccines:**

   "Today, (name of child), is due for 3 vaccines: a TDap booster, a vaccine for meninigitis, and the first of 2 doses for HPV vaccine."

   Parent agrees ➡ Give vaccine and book the follow-up appointment for the remaining dose (patients age 15 and older will require 2 additional doses).

2. If parent hesitates about HPV, or about having 3 vaccines at once; identify concerns about HPV and address:

   A. **Why now?** "HPV vaccine prevents cancers caused by HPV viruses. Just like any other vaccine, it only works if given before exposure to the virus occurs. The risks of HPV exposure increase after age 13. So we give it before 13."

   B. **Is it safe?** "Yes. It's been extensively tested and proved safe - and it works. In the U.S., more than 120 million doses have been given and more than 270 million worldwide."

   C. "I've had my son/daughter vaccinated (if true)" OR "If it was my son/daughter, I would have him/her vaccinated."

   Parent agrees ➡ Give vaccine and book the follow-up appointment for the remaining dose.

3. If parent still hesitates, provide brochure, circle CDC website for more information and get verbal or written agreement for follow-up:

   "I strongly recommend this vaccine for all 11 and 12 year olds. We'll talk about this again next time you're in the office. We don't want to miss the chance to prevent cancer for (name of child)."

Children's HOSPITAL • ST. LOUIS BJC HealthCare

Washington University in St.Louis SCHOOL OF MEDICINE

© 2026 Wang R et al. *JAMA Network Open*.

eTable 3. Number of Clinicians and Patients by Practice at 12, 24, and 36 Months[b]

Practice Code	12 months		24 months		36 months	
**Control**	**Clinicians (n=39)**	**Patients (n=1538)**	**Clinicians (n=39)**	**Patients (n=1615)**	**Clinicians (n=35)**	**Patients (n=1410)**
P10[a]	3	123	3	111	-	-
P12	2	119	2	119	2	109
P14	3	132	3	136	2	118
P15	5	242	5	266	5	256
P22	1	33	1	44	1	31
P25	5	269	5	295	5	291
P26	5	222	5	223	5	214
P27	3	69	3	75	3	65
P28	10	266	10	292	10	279
P29	2	63	2	54	2	47
**Intervention**	**Clinicians (n=45)**	**Patients (n=2141)**	**Clinicians (n=44)**	**Patients (n=2151)**	**Clinicians (n=43)**	**Patients (n=2211)**
P11	4	215	4	240	4	240
P13	4	161	4	161	4	161
P16	4	220	4	240	4	238
P17	4	176	4	185	4	205
P18	4	224	4	219	4	213
P19	5	245	5	263	5	284
P20	6	231	5	203	4	165
P21	8	435	8	381	8	418
P23	4	141	4	169	4	187
P24	2	93	2	90	2	100

a. Practice 10 was lost to follow-up at 36 months.
b. Corresponding to the CONSORT diagram.

© 2026 Wang R et al. *JAMA Network Open.*

eTable 4. Intervention Effects on HPV Vaccination Initiation and Completion by Study Year: Percentage Point Differences[a]

Timepoint	HPV vaccination initiation PP difference (95% CI)			HPV vaccination completion PP difference (95% CI)		
	Unadjusted	Model 1[b]	Model 2[c]	Unadjusted	Model 1[b]	Model 2[c]
12 months	0.8 (-9.2 to 10.9)	0.6 (-6.2 to 7.4)	0.5 (-7.6 to 8.6)	3.8 (-10.0 to 17.6)	4.0 (-3.8 to 11.8)	1.4 (-5.7 to 8.4)
24 months	3.1 (-6.5 to 12.7)	2.7 (-3.8 to 9.2)	2.7 (-5.0 to 10.5)	1.8 (-12.1 to 15.8)	1.9 (-5.9 to 9.7)	-0.9 (-8.1 to 6.3)
36 months	6.6 (-3.0 to 16.1)	6.2 (-0.4 to 12.8)	6.9 (-0.9 to 14.7)	8.3 (-5.6 to 22.2)	8.6 (0.7 to 16.4)	5.4 (-2.0 to 12.7)
$P_{\text{Group x Year}}$	.04	.03	.04	.04	.04	.05

Abbreviations: CI, confidence interval; HPV, human papillomavirus; PP, percentage point.

[a] Results from mixed effects logistic regression with practice and clinician as random effects. Results show intervention minus control group at 12 months, 24 months (end of intervention), and 36 months (post-intervention). Intention-to-treat analysis.

[b] Adjusted for clinician baseline HPV vaccination rate. HPV vaccination initiation $ICC_{practice}$=0.03, $ICC_{clinician}$=0.03; HPV vaccination completion $ICC_{practice}$=0.02, $ICC_{clinician}$=0.03.

[c] Additionally adjusted for patient gender, race, insurance, and clinician sex.

© 2026 Wang R et al. *JAMA Network Open.*

eFigure 2. HPV Vaccination Initiation and Completion by Patient and Clinician Characteristics[a]

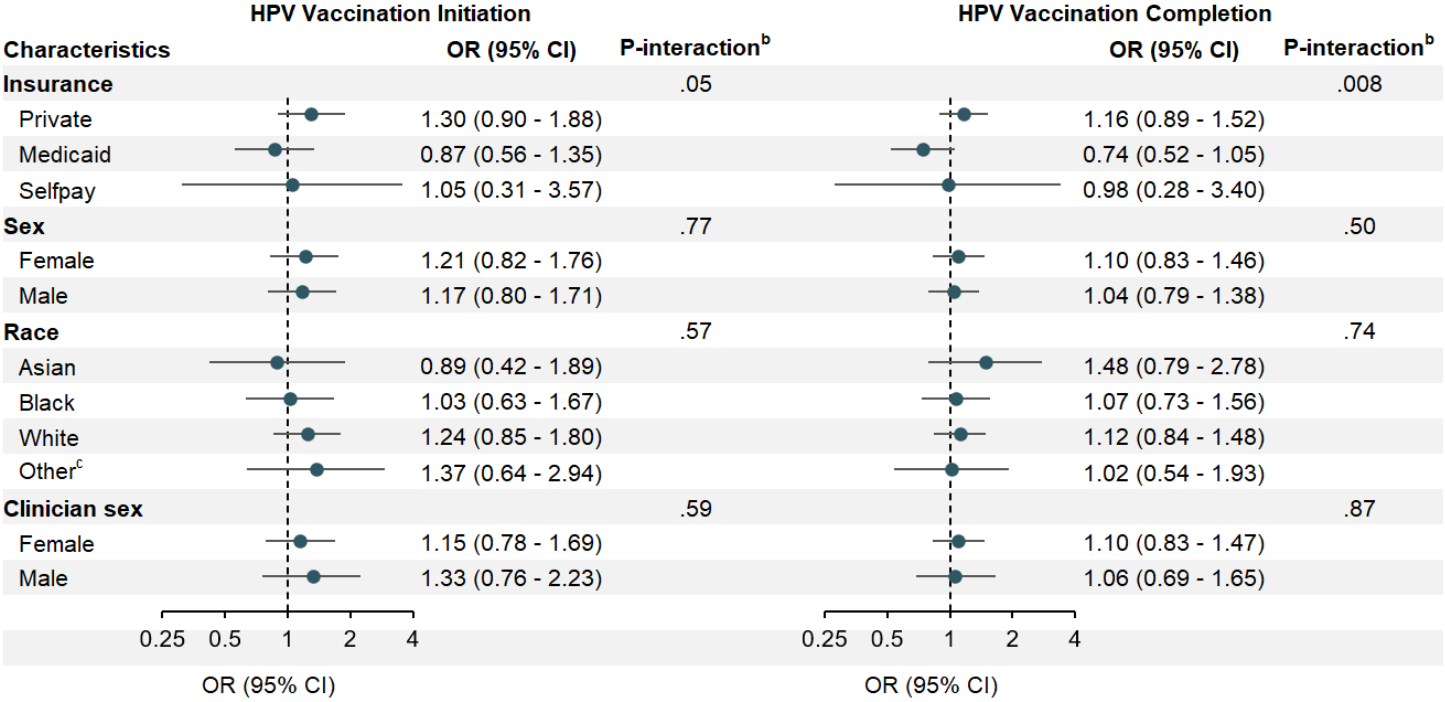

Characteristics	HPV Vaccination Initiation OR (95% CI)	P-interaction[b]	HPV Vaccination Completion OR (95% CI)	P-interaction[b]
**Insurance**		.05		.008
Private	1.30 (0.90 - 1.88)		1.16 (0.89 - 1.52)	
Medicaid	0.87 (0.56 - 1.35)		0.74 (0.52 - 1.05)	
Selfpay	1.05 (0.31 - 3.57)		0.98 (0.28 - 3.40)	
**Sex**		.77		.50
Female	1.21 (0.82 - 1.76)		1.10 (0.83 - 1.46)	
Male	1.17 (0.80 - 1.71)		1.04 (0.79 - 1.38)	
**Race**		.57		.74
Asian	0.89 (0.42 - 1.89)		1.48 (0.79 - 2.78)	
Black	1.03 (0.63 - 1.67)		1.07 (0.73 - 1.56)	
White	1.24 (0.85 - 1.80)		1.12 (0.84 - 1.48)	
Other[c]	1.37 (0.64 - 2.94)		1.02 (0.54 - 1.93)	
**Clinician sex**		.59		.87
Female	1.15 (0.78 - 1.69)		1.10 (0.83 - 1.47)	
Male	1.33 (0.76 - 2.23)		1.06 (0.69 - 1.65)	

Abbreviations: HPV, human papillomavirus; CI, confidence interval; OR, odds ratio;

a. Comparison of intervention vs. control group. The model was adjusted for group-by-time interaction term, clinician baseline HPV vaccination rate, all covariates except the subgroup variable being analyzed (patient sex, race, insurance, and clinician sex).

b. P-interaction values test whether the intervention effect differs by subgroup, adjusted for time.

c. Includes American Indian/Alaskan Native, and Native Hawaiian/Pacific Islander, and multiracial.

© 2026 Wang R et al. *JAMA Network Open*.

eTable 5. Clinician Characteristics Between Responders and Nonresponders Within Each Group[a]

Timepoint	Clinician Characteristics	Control, No. (%)			Intervention, No. (%)		
		Responders (n=23)	Non-responders (n=16)	*P*	Responders (n=37)	Non-responders (n=7)	*P*
12 months	**Sex**			.67			.004
	Female	20 (87)	13 (81)		28 (76)	1 (14)	
	Male	3 (13)	3 (19)		9 (24)	6 (86)	
	**Years in practice, median (IQR)**	18 (7-23)	24 (16-29)	.16	16 (10-21)	25 (23-38)	.01
		**Responders (n=26)**	**Non-responders (n=12)**	***P***	**Responders (n=36)**	**Non-responders (n=7)**	***P***
24 months	**Sex**			.15			.19
	Female	20 (77)	12 (100)		26 (72)	3 (43)	
	Male	6 (23)	0 (0)		10 (28)	4 (57)	
	**Years in practice, median (IQR)**	19 (10-24)	19 (8-27)	.90	17 (11-21)	25 (18-38)	.14

Abbreviations: IQR, interquartile range

[a.] Survey numbers reflect distribution after each 12-month intervention period; clinicians who left before survey distribution were not surveyed.

© 2026 Wang R et al. *JAMA Network Open.*

eReferences.

1. Petca A, Borislavschi A, Zvanca ME, Petca RC, Sandru F, Dumitrascu MC. Non-sexual HPV transmission and role of vaccination for a better future (Review). *Exp Ther Med*. Dec 2020;20(6):186. doi:10.3892/etm.2020.9316
2. American Cancer Society. HPV (human papillomavirus). Accessed August 6, 2025. https://www.cancer.org/cancer/risk-prevention/hpv.html

© 2026 Wang R et al. *JAMA Network Open.*
