# Supplementary material for: A Multicomponent Strategy to Increase Human Papillomavirus Vaccination Rates in Primary Care: A Cluster Randomized Clinical Trial
Source: JAMA Netw Open. 2026 Feb 26;9(2):e260049. doi: 10.1001/jamanetworkopen.2026.0049 (PMC12947024; doi:10.1001/jamanetworkopen.2026.0049)
Supplement: Supplement 3. — Data Sharing Statement [file jamanetwopen-e260049-s003.pdf]

Data Sharing Statement

Wang. A Multicomponent Strategy to Increase Human Papillomavirus Vaccination Rates in Primary Care. *JAMA Netw Open*. Published February 26, 2026.
doi:10.1001/jamanetworkopen.2026.0049

Data
Additional Information: ClinicalTrials.gov; https://clinicaltrials.gov;NCT04180462
Data available: No

Additional Information
Explanation for why data not available: The data used in this study were collected from 20 pediatric practices. Given that explicit permission for data sharing was not obtained from these practices, the data cannot be shared openly. However, we are committed to transparency and reproducibility in research. Researchers interested in accessing the data can contact the corresponding author to discuss potential avenues for data sharing. Any data sharing will be subject to approval from the pediatric practices involved and adherence to ethical and legal guidelines to protect patient confidentiality and comply with institutional and regulatory requirements.
